# Unmasking the Aliphatic Repertoire: New Polyunsaturated Metabolites in *Bupleurum falcatum sensu lato* Provide Chemotaxonomic Insights

**DOI:** 10.3390/plants14101432

**Published:** 2025-05-10

**Authors:** Milica D. Nešić, Milan S. Nešić, Irena Lj. Raca, Miha Bukleski, Niko S. Radulović

**Affiliations:** 1 Department of Chemistry, Faculty of Sciences and Mathematics, University of Niš, Višegradska 33, 18000 Niš, Serbia; milica.stevanovic992@gmail.com (M.D.N.); milan.nesic@pmf.edu.rs (M.S.N.); 2 Department of Biology and Ecology, Faculty of Sciences and Mathematics, University of Niš, Višegradska 33, 18000 Niš, Serbia; irena.raca@pmf.edu.rs; 3 Institute of Chemistry, Faculty of Natural Sciences and Mathematics, Ss. Cyril and Methodius University, Arhimedova 5, 1000 Skopje, North Macedonia; mihabukleski@yahoo.com

**Keywords:** *Bupleurum falcatum*, polyunsaturated, extract, chemotaxonomy

## Abstract

*Bupleurum falcatum* (Apiaceae) *sensu lato* includes multiple infraspecific taxa with longstanding taxonomic ambiguities, often resulting from incomplete morphological and chemical characterizations. Herein, diethyl ether extracts were analyzed from four Balkan populations that were tentatively identified as *B. falcatum* subsp. *falcatum* (*syn*. *B. falcatum*) and *B. falcatum* subsp. *cernuum* (*syn*. *B. sibthorpianum*). Comprehensive chromatography and spectroscopic techniques (GC-MS and 1D/2D NMR) enabled the isolation of several newly identified aliphatic polyunsaturated esters, including compounds bearing uncommon conjugated tetraene and triyne backbones. These novel structures differ from canonical falcarinol derivatives by lacking the usual 3-hydroxylation, suggesting a divergent branch in the crepenynate pathway. The chemical profiles of each sample correlated closely with leaf morphology and infraspecific designations: for example, the Galičica Mt. population of *B. falcatum* featured a unique newly detected heptadecadientriyne, while the populations from Šar Planina and Suva Planina displayed distinct polyunsaturated repertoires. Extracts from *B. sibthorpianum* likewise contained stereoisomeric compounds that highlight metabolic divergence. Collectively, these findings demonstrate significant chemotypic variation within the *Bupleurum falcatum* complex and provide the first account of less-polar secondary metabolites, including newly discovered polyunsaturated metabolites. Future research integrating molecular markers and bioactivity assays may elucidate how these specialized metabolites contribute to both the taxonomy and pharmacological potential of these understudied taxa.

## 1. Introduction

The genus *Bupleurum* L. comprises approximately 248 species, widely distributed across the Northern Hemisphere, Eurasia, and North Africa, and is known for its medicinal properties [1]. *Bupleurum falcatum* L. (Apiaceae), a perennial herb native to East Asia (China, Japan, and Korea), has a long history of use in traditional medicine, particularly in Chinese herbal practices, where it is valued for its therapeutic effects, including anti-inflammatory, fever-reducing, and pain-relieving properties [2]. The taxonomy and nomenclature hiding behind the name *B. falcatum sensu lato*/*stricto*, as well as its questionable synonymy/relationship to *B. sibthorpianum sensu lato*/*stricto*, present significant challenges due to inconsistencies across regional flora [3,4,5].

In the book *Flora of the Socialist Republic of Serbia* [3], *B. falcatum* L. and *B. sibthorpianum*, described by Smith in a work co-authored by Sibth. and Sm., are recognized as separate species. *Bupleurum falcatum* is further characterized by a specific form, *B. falcatum* f. *latifolium* Schur, while *B. sibthorpianum* is noted as a highly variable taxon, encompassing the *B. sibthorpianum* var. *sibthorpianum*, *B. sibthorpianum* var. *orbelicum* (Vel. Hayek), and *B. sibthorpianum* var. *diversifolium* (Roch.) Hayek. Key morphological distinguishing features include *B. falcatum* having longer petiolate basal leaves that are elliptic or elongated, whereas *B. sibthorpianum* is characterized by sedentary, narrower linear-lanceolate basal leaves [3]. In the book *Flora of the Republic of Macedonia* [4], *B. falcatum* is divided into two subspecies: *B. falcatum* subsp. *falcatum* and *B. falcatum* subsp. *cernuum* (Ten.) Arcangeli (*syn*. *B. sibthorpianum*). The former is characterized by elliptical to elongated basal leaves with long petioles, while the latter has linear, sessile, or short-petiolate basal leaves. Varieties such as *B. falcatum* var. *orbelicum* Vel. and *B. falcatum* var. *montenegrinum* (Wollf.) Hay are also mentioned, though detailed morphological descriptions are lacking [4]. In the book *Flora Europaea* [5], the same two subspecies of *B. falcatum* are described with their key morphological differences: *B. falcatum* subsp. *falcatum* having distinctly petiolate, elliptical to oblong leaves and *B. falcatum* subsp. *cernuum* with sessile, linear leaves. The variability in species and infraspecific classification reflects both morphological diversity and historical taxonomic interpretations, highlighting the need for a thorough re-examination.

The pharmacologically significant chemical constituents of *B. falcatum* have been well documented, particularly its triterpenoid saponins, such as saikosaponins A, C, and D, which represent the major bioactive compounds [6]. These saponins have been shown to exhibit potent anti-inflammatory, hepatoprotective, antitumor, and immunomodulatory effects [2,6]. Although primarily concentrated in the roots, saikosaponins are also present in the stem and leaves, with their levels varying in response to environmental and developmental factors [1,6]. Additionally, polyacetylenes like saikodiyne A, B, and C, along with sterols (e.g., *β*-sitosterol, stigmasterol), alkaloids, and glycosides, contribute to the plant’s complex chemical profile and diverse pharmacological actions [1,6].

The volatile oil composition of *B. falcatum* is predominantly composed of aldehydes and alkenes, with key compounds including (2*E*,4*E*)-2,4-decadienal and *β*-caryophyllene [7,8,9,10,11]. Variability in composition is observed among different plant parts. Studies on the aerial parts by Abolfazl et al. identified torilenol (39.1%), spathulenol (19.6%), and *α*-cubebene (8.1%) as the predominant compounds [7,8]. Rustaiyan et al. reported a higher concentration of *α*-pinene (29.4%) and spathulenol (27.7%) [9].

*Bupleurum falcatum* subsp. *cernuum* has been less extensively studied; nonetheless, its volatile profile resembles that of *B. falcatum* [10], while targeted analyses have revealed the presence of saikosaponins (b2, b3, b4) and of the flavonoid glycosides rutin, avicularin, and guaijaverin [6,12]. This variation in the chemical composition within the *B. falcatum* subspecies reflects the complexity of its pharmacological potential and the role of genetic and environmental factors in shaping its bioactive compound profiles.

Having collected several samples of *B. falcatum* from different locations in the Balkans, potentially representing different taxonomic variants within the *B. falcatum* taxon, the aim was set to investigate the chemical composition of diethyl ether extracts from the aboveground parts of these taxa. Moreover, the study was focused on providing the first chemical data for the hypothesis that distinct chemical races or taxonomic differences exist within *B. falcatum*. Extracts of *B. falcatum* underwent comprehensive chromatographic separations, and the structures of the isolated constituents were elucidated by spectroscopic means and chemical transformations. Structural elucidation and complete NMR spectroscopic assignments were conducted using iterative ^1^H NMR full spectral simulations. This study serves as an initial investigation into the chemical divergence between *B. falcatum* and *B. sibthorpianum*, laying the groundwork for future research aimed at understanding the implications of these differences for the pharmacological properties of these taxa.

Notably, many accounts of *B. falcatum* focus on polar components, such as saikosaponins. Far fewer studies address less-polar constituents, which may be equally relevant for chemotaxonomic differentiation and biological activity. By investigating diethyl ether extracts, the present work aims to uncover hitherto unexplored metabolites, thereby broadening the known chemical diversity in *B. falcatum sensu lato* and identifying potential lineage-specific markers.

## 2. Results and Discussion

Motivated by the lack of chemical data on the less polar constituents of *B. falcatum*—in contrast to the well-documented highly polar metabolites—and building on findings from other *Bupleurum* taxa [13,14], diethyl ether extracts from several accessions of *B. falcatum sensu lato* were herein investigated with the aim of identifying potentially new natural compounds. Chromatographic separations on SiO_2_ afforded three entirely new chemical entities and an additional metabolite previously unreported in plants. The structural elucidation of these compounds is presented and discussed first, followed by an analysis of the remaining composition. Among the identified secondary metabolites, compounds **1**, **2**, and **8** are characterized as entirely new aliphatic polyunsaturated compounds. Although similar conjugated polyunsaturated compounds have been reported in *Bupleurum* taxa and other members of the Apiaceae [1,6,15], the presence of distinct variants in *B. falcatum* is confirmed here for the first time. While similar conjugated polyunsaturated compounds have been reported in *Bupleurum* taxa and other Apiaceae members [1,6,15], the data presented herein confirm the presence of distinct variants in *B. falcatum* for the first time.

**Figure 1:**
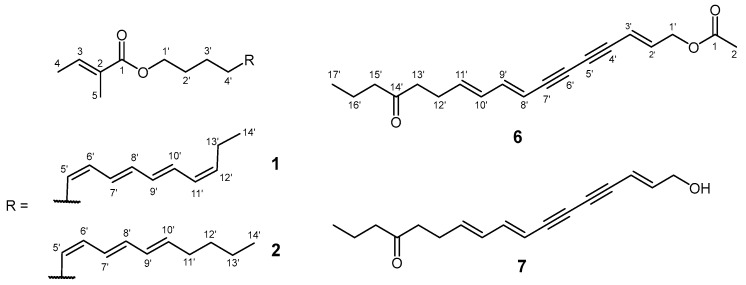
Structures of compounds **1**, **2**, and **6**, isolated from BFS extract, and compound **7**.

A 415-mg fraction of the *B. falcatum* extract from Suva Planina Mountain (BFS), eluted with hexane-Et_2_O (6:1, *v*/*v*) and containing compound **1** (Figure 1), 10-nonacosanone, and minor components, was rechromatographed on silica gel to yield 30 mg of pure compound **1**. EIHRMS disclosed the molecular formula of **1** to be C_19_H_28_O_2_ (Δ = +0.5 mmu). The fragmentation pattern observed in the mass spectrum of **1** (*m*/*z* 91, 105, 117, and 131) was consistent with that of praealtaesters previously isolated from *B. praealtum* [13]. Based on this similarity, it was presumed that compound **1** is likely an ester of the same polyunsaturated alcohol, 5,7,9,11-tetradecatetraenol. The difference between the molecular ion of compound **1** (*m*/*z* 288) and the molecular mass of the aforementioned alcohol, combined with the presence of a fragment ion at *m*/*z* 83 in the mass spectrum of **1**, suggested that it is an ester of the 5,7,9,11-tetradecatetraenol and an unsaturated five-carbon acid.

The ^13^C NMR spectrum of compound **1** displayed 19 peaks, including one acyl carbon signal at *δ*_C_ 168.3 (C-1) and a signal of an oxygenated sp^3^-hybridized carbon at *δ*_C_ 64.4 (C-1′). The signals of the methylene C-1′ hydrogens were observed at *δ*_H_ 4.13 in the ^1^H NMR (Table 1) and correlated through three bonds with C-1 (Figure S1, Supplementary Material). The ^1^H NMR spectrum (Figure S2, Supplementary Material) is displayed, along with the signals of two methyl groups (*δ*_H_ 1.78 and 1.83, Table 1) from the acid part of the molecule, one additional methyl group signal as a non-virtually coupled triplet at *δ*_H_ 1.01 (*J* = 7.5 Hz), implying that the unsaturated functionalities were near the *ω*-end of the alcohol, and that **1** did not contain any further branching. All four unsaturations were located in four double bonds, as concluded from the 8 sp^2^-CH ^13^C NMR/DEPT-90 signals (Table 1), which appeared to be mutually conjugated based on UV. Their positions in the chain were straightforwardly determined to be between carbons C-5′ and C-6′, C-7′ and C-8′, C-9′ and C-10′, and C-11′ and C-12′ from gHMBC (Figure S3, Supplementary Material) and the gradient ^1^H-^1^H COSY spectra, i.e., there is an ethyl group on the *ω*-end of the alcohol. The geometries of the C-5′–C-6′ and C-11′–C-12′ double bonds were concluded to be *Z*, based on the observable proton–proton (*J*_H-5′–H-6′_ = *J*_H-11′–H-12′_ = 11.0 Hz). A comparison with the NMR data on the closely related praealtaesters further corroborated the (5*Z*,7*E*,9*E*,11Z) configuration of **1**. The observed NOESY and ROESY interactions of **1** support the assigned configurations of the double bonds. However, due to the very similar chemical shifts of the inner double bonds, not all expected cross-peaks were clearly distinguishable. For these protons, spin simulations played a crucial role in confirming the stereochemistry by accurately reproducing the observed coupling patterns. The combined data are consistent with and supportive of the proposed configurations and are in agreement with related precedents in the literature [13]. Additionally, the diagnostic ^13^C NMR shifts of the allylic methylenes at C-4′ and C-13′ agreed well with those in veronaester, having the (5*Z*,7*E*,9*E*,11Z) configuration [13]. While, as expected, the corresponding shifts of praealtaester A, which contains the (5*Z*,7*E*,9*E*,11*E*)-5,7,9,11-tetradecatetraenyl group [13], only significantly differed for C-13′ by cca.–4 ppm.

Within the TIC chromatogram of the same BFS extract fraction containing compound **1**, an additional peak with a similar fragmentation pattern in its mass spectrum was observed. Notably, the molecular ion was detected at *m*/*z* 290, which was, based on EIHRMS, concluded to correspond to C_19_H_30_O_2_, suggesting the compound has one fewer double bond. The fragmentation ion at *m*/*z* 83 further supports its identification as an ester of tiglic acid, with the alcohol moiety containing one less degree of unsaturation. The subsequent purification steps yielded a fraction enriched in compound **2** (Figure 1). The analysis of the NMRs of this enriched fraction was not a straightforward task as there was signal overlap in the region of the double-bond protons, and it did not allow an unambiguous conclusion. The double bonds in compound **2** were tentatively positioned at C-5′, C-7′, and C-9′, based on the unobstructed HMBC interactions, but these needed to be confirmed and the stereochemistry determined. To resolve this, a model compound was synthesized that possesses the assumed regiochemistry of the three double bonds to aid in the configurational assignment.

**Figure 2:**
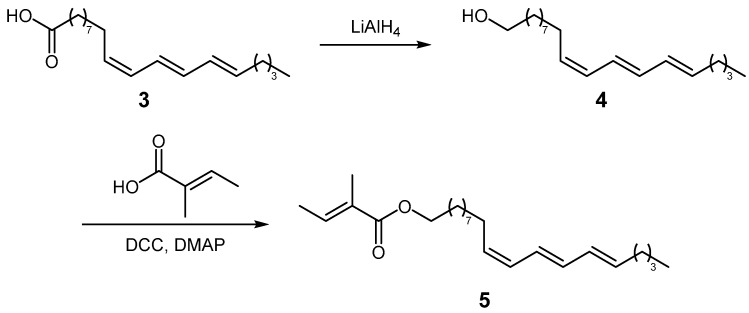
Synthesis of (9*Z*,11*E*,13*E*)-octadeca-9,11,13-trien-1-yl tiglate (**5**).

**Figure 3:**
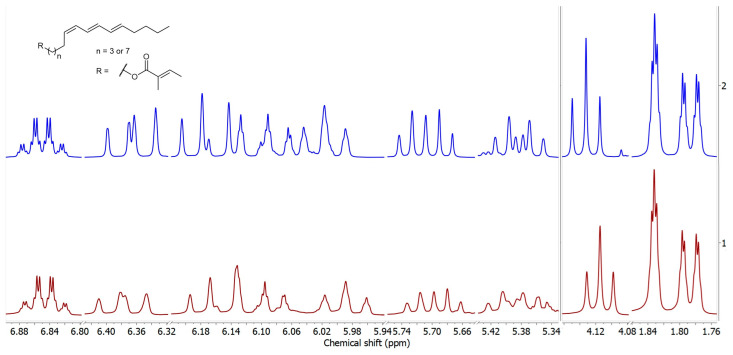
Selected expansions of ^1^H NMR spectrum of compound **2** (up) and compound **5** (down).

*α*-Eleostearic acid (**3**), (9*Z*,11*E*,13*E*)-9,11,13-octadecatrienoic acid, featuring three conjugated double bonds having the same tentatively assigned (*ω*-5)-regiochemistry as in compound **2**, can be readily attained from commercial tung oil (*Vernicia fordii* (Hemsl.) Airy Shaw, Euphorbiaceae) [16]. The reduction of tung oil with LiAlH_4_ gave a tetrahomo-alcohol (**4**, Figure 2), which was further esterified with tiglic acid via *Steglich* esterification, yielding the corresponding homologous ester ((9*Z*,11*E*,13*E*)-octadeca-9,11,13-trien-1-yl tiglate, **5**). A detailed analysis of this tiglate permitted us to ascertain the configuration of the double bonds. Further, the NMR spectra of the synthesized model of compound **5** and the fraction enriched with compound **2** were compared to confidently confirm the presence and position of the three conjugated double bonds in the alcohol moiety of the ester, as well as to affix the 5*Z*,7*E*,9*E* configuration to compound **2** (Figure 3). Additionally, the expected difference of ca. 400 units between the retention indices of the two compounds (RI 2652 for compound **5** and 2253 for compound **2**) allowed the positive identification of (5*Z*,7*E*,9*E*)-tetradeca-5,7,9-trien-1-yl (*E*)-2-methylbut-2-enoate in the BFS extract.

A more polar fraction (hexane-Et_2_O = 3:2, *v*/*v*, 259 mg) of the BFS extract represented a mixture containing compound **6** (Figure 1), which, based on its fragmentation pattern (dominant ions at *m*/*z* 43, 71, and 115 and a molecular ion at *m*/*z* 298), was presumed to be the acetate of compound **7**. Its molecular formula was inferred to be C_19_H_22_O_3_ from EIHRMS. Similarly for **7** [17,18] (vide infra), a ketone carbon was observed at *δ*_C_ 209.8 in the ^13^C NMR spectrum, positioned at C-14′ from the *m*/*z* 71 fragment, and the coupling constants of protons H-2′ and H-3′ (15.8 Hz) confirmed the *E*-configuration of the double bond. The HMBC interaction of the protons at position 1′ with the ester carbon C-1 at *δ*_C_ 170.4 further confirmed the compound’s identity as the acetate. The spectral data for compound **6** are provided in Sommerwerk et al. [19], and here it is reported for the first time as a new natural product.

**Figure 4:**
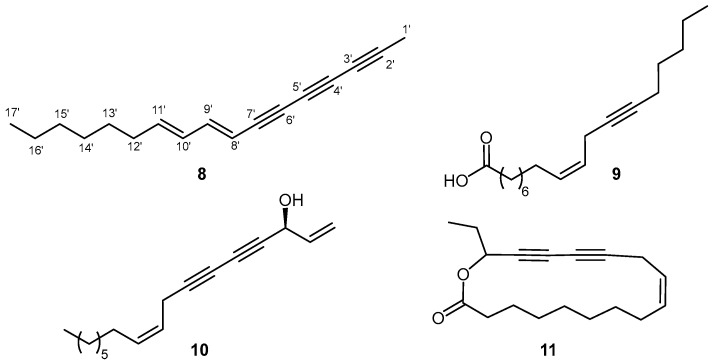
Structures of compound **8**, crepenynic acid (**9**), (*Z*)-falcarinol (**10**), and homodihydrofalcarinolide (**11**).

The dry flash chromatography of the extract of *B. falcatum* from Mt. Galičica (BFG) yielded 25 fractions. Fraction 9, eluted with hexane:Et_2_O 13:1 (41 mg), contained compound **8** (Figure 4), which displayed a molecular ion at *m*/*z* 224 and fragment ions at *m*/*z* 139, 152, and 165 in its mass spectrum, which is indicative of a high degree of unsaturation (equivalent to propenylnaphthalene or a tetrayne). The molecular formula of **8**, in agreement with the proposed number of unsaturations, was deduced to be C_17_H_20_ from its EIHRMS (Δ = +0.5 mmu). In the ^13^C NMR spectrum, 17 signals were observed, and the analysis of the DEPT spectra revealed the presence of four sp^2^ C-H signals (Table 2). The coupling constants between the protons attached to these carbons, H-8 and H-9, as well as H-10 and H-11, measured at approximately 15.6 and 15.1 Hz, respectively, indicating that the configurations of the double bonds were *trans*. Furthermore, the coupling constant between H-9 and H-10 suggested that the two double bonds adopt an *s*-*trans* conformation. The DEPT spectrum also revealed the presence of seven sp^3^-hybridized carbon atoms: five methylene and two methyl groups, one of which displayed an unusually low chemical shift at *δ*_C_ 4.86. The existence of six non-protonated carbons in the chemical shift range of *δ*_C_ 59.5–78.6 was also noted. These signals were recognized as belonging to sp-hybridized carbon atoms in three triple bonds.

The carbon at *δ*_C_ 4.86 that demonstrated a characteristic shift of a highly shielded propargyl methyl was associated with protons exhibiting a chemical shift of *δ*_H_ 1.99 (Table 2), and their HMBC interactions with signals at *δ*_C_ 78.6 and 65.2 (corresponding to carbons C-2 and C-3) unequivocally confirmed that this methyl group is directly attached to a sp-hybridized carbon. Additional, albeit weaker, HMBC correlations of these protons with carbons at *δ*_C_ 68.0 and 59.50 suggested that these are the mutually triple-bonded carbons C-4 and C-5, respectively. Additionally, the interactions of protons H-9 and H-8 with carbons C-7 and C-6, as well as “weaker” interactions with C-5 and C-4 in the HMBC spectrum, along with a comparison with the available data in the literature [20,21,22], enabled the assignment of the signals to these six sp-hybridized carbons. Thus, the compound was identified as (8*E*,10*E*)-heptadeca-8,10-dien-2,4,6-triyne, a new compound isolated from the *B. falcatum* from Mt. Galičica.

**Figure 5:**
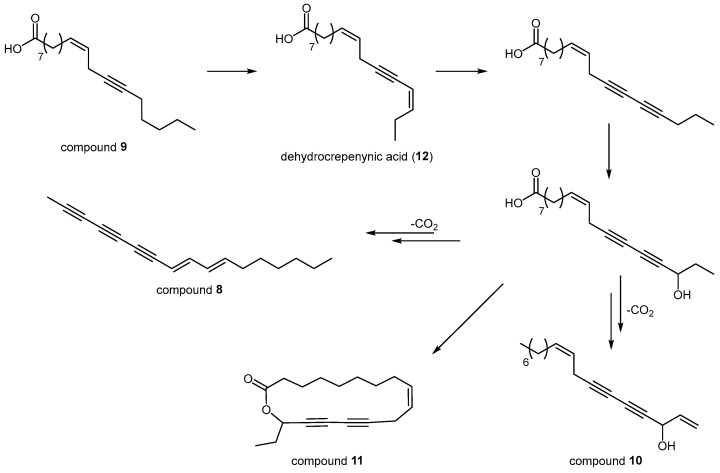
Proposed biosynthetic pathway to compound **8** and related falcarinol-type polyacetylenes via the crepenynate pathway.

It can be speculated that the biosynthesis of compound **8** and homodihydrofalcarinolide (**11**, Figure 4), previously identified in *B. veronense* Turra [13], follows the crepenynate pathway in a manner analogous to falcarinol-type polyacetylenes [23,24]. In this pathway, oleic acid undergoes sequential desaturation and acetylenation to form linoleic acid, crepenynic acid (**9**), and dehydrocrepenynic acid (**12**), key intermediates in route to polyacetylene scaffolds [23,25]. For falcarinol (**10**) and homodihydrofalcarinolide, a 17,18-didehydro acid intermediate is oxidized, hydroxylated, and ultimately decarboxylated to afford the final falcarinol framework [23] (Figure 5).

Based on the herein given structural analyses, a biosynthetic route leading to compound **8** could be suggested that diverges following the hydroxylation step responsible for generating falcarinol’s characteristic 3-hydroxy moiety (Figure 5). This would give rise to its distinct structure and implicates compound **8** as either an unrecognized intermediate within the falcarinol series or a branch point for structurally related derivatives. Such a scenario highlights the inherent complexity and potential variability within the crepenynate pathway, which appears to accommodate multiple oxidation and cyclization options even within a single plant genus.

A notable parallel to these findings can be seen with the structurally related pentadeca-8,10-dien-2,6,4-triyne isolated from *Jungia spectabilis* D.Don (Asteraceae) [26]. Since *Jungia* species typically display a different profile of terminal substituents on their polyacetylenes than the falcarinol-like series, their biosynthesis may follow a distinct, “non-falcarinol” route [26]. Taken together, these observations of homologous C_15_ and C_17_ polyacetylenes across both Apiaceae and Asteraceae suggest that convergent, yet subtly divergent, pathways operate in different plant families, underscoring the biochemical plasticity of acetylenic lipid metabolism.

Although the biological activities of saikosaponins in *B. falcatum* have been extensively studied, much less is known about the pharmacological roles of these newly isolated polyunsaturated derivatives. Previous work on structurally related falcarinol-type polyacetylenes in other Apiaceae species suggests possible anti-inflammatory and anticancer potential [1]. Further studies on these new *Bupleurum* compounds, including cell-based assays or in vivo models, could help clarify how their presence might contribute to the medicinal properties historically attributed to *B. falcatum*.

**Figure 6:**
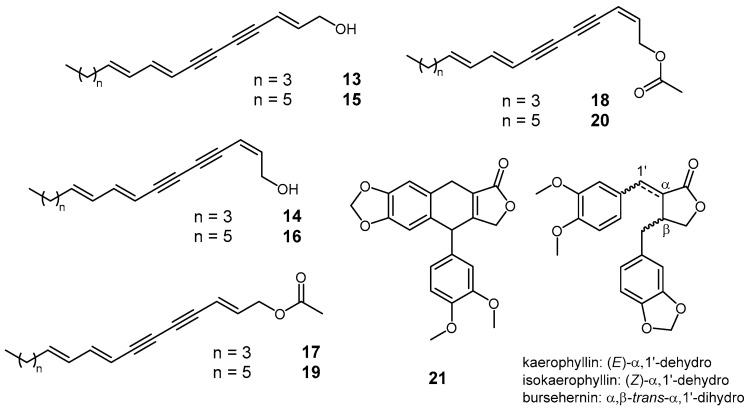
Structures of compounds **13**–**21** found in the BFS extract and the lignans detected in the BFG extract.

The initial GC-MS analysis of the crude diethyl ether extract of BFS identified 10-nonacosanone (15.3%) as the predominant volatile constituent (under GC conditions), along with germacrene D (4.1%), long-chain aldehydes (hexacosanal 6.0%, octacosanal 4.1%), alcohols (1-hexacosanol 9.8%), and *n*-alkanes (Table 3). The gradient dry-flash chromatography of the extract yielded 30 fractions, while the identification of compounds **1**, **2,** and **6** from fractions 11 and 18 was already described. Fraction 10 (hexane-Et_2_O 12:1, *v*/*v*, 510 mg) primarily contained long-chain aldehydes and methyl esters of fatty acids. Fraction 20, eluted with hexane-Et_2_O (1:4, *v*/*v*, 168 mg), was identified as pure (2*E*,8*E*,10*E*)-pentadecatriene-4,6-diyn-1-ol (**13**, Figure 6), as confirmed by the NMR analysis, with the spectral data being consistent with previous reports [27,28]. The preceding fraction (19, hexane-Et_2_O 3:2, *v*/*v*, 340 mg) comprised a mixture of (2*E*,8*E*,10*E*)- and (2*Z*,8*E*,10*E*)-pentadecatriene-4,6-diyn-1-ols (**13** and **14**, Figure 6), along with bupleurynol and its stereoisomer (**16** and **15**, Figure 6) [29], as determined by mass spectrometry and retention index comparisons (see Section 3). In a less polar fraction (13, hexane-Et_2_O 4:1, *v*/*v*, 306 mg), four compounds were detected, all exhibiting a dominant fragment ion at *m*/*z* 43 and a fragmentation pattern identical to compounds **13**–**16**. Based on these features and their molecular ions, they were tentatively identified as acetate derivatives of the corresponding alcohols. To confirm this, fraction 19 was subjected to a *Steglich* esterification reaction with acetic acid, and the retention indices and mass spectra of the synthesized esters were compared with those of the detected compounds in fraction 13. This analysis confirmed that the observed compounds were indeed the acetate derivatives of alcohols **13**–**16** (**17**–**20**, Figure 6). Fraction 26 (EtOAc, 20 mg) was identified as a pure lignan (**21**, Figure 6) that was previously reported in *Bupleurum handiense* (Bolle) G.Kunkel [30]. Compound **7** (Figure 1) was detected in fraction 23 (Et_2_O, *v*/*v*, 170 mg) as part of a complex mixture. Its identity was inferred from mass spectral data and subsequently confirmed by an NMR analysis. The ^13^C NMR spectrum of fraction 23 displayed a signal at *δ*_C_ 203.5, indicative of a ketone, while the ^1^H NMR spectrum showed signals at *δ*_H_ 5.5 and 5.7 with a coupling constant of 15 Hz, confirming the *E*-configuration of the double bond at position 2.

Comparative analyses of the diethyl ether extracts from the four *Bupleurum* taxa (BFS, BFK, BFG, and BS, Table 3) demonstrate marked phytochemical diversity, aligning with earlier accounts of morphological and taxonomic complexity [3,4]. Overall, these data reinforce the notion that variations in metabolite profiles can reflect underlying genetic and environmental factors that shape distinct chemotypes.

The chemotaxonomic profile, characterized by relatively high levels of 10-nonacosanone, 1-hexacosanol, hexacosanal, and compound **1**. The distinction suggests that this sample most likely corresponds to *B. falcatum* f. *latifolium* reportedly restricted to Suva planina Mt., as noted by Nikolić [3]. Although Micevski [4] posited that *B. falcatum* subsp. *cernuum* is the prevailing entity on Šar planina Mt., the BFK sample from this locality shows chemical features suggesting a mixture of morphotypes. Most individuals align with *B. falcatum* subsp. *falcatum*, but at least one individual shows features consistent with *B. falcatum* subsp. *cernuum*, indicating the possibility of sympatric or transitional populations. This morphological ambiguity mirrors the diverse metabolic traits observed in the BFK extract, notably the dominance of compounds **13** and **17**, paired with fewer long-chain alkanes and alcohols, compared to the BFS extract. Additionally, the sample collected from Olympus Mt. was characterized by the highest content of 10-nonacosanone among all the analyzed samples, further emphasizing its distinct chemical profile.

Meanwhile, the BFG extract from Galičica Mt. demonstrates chemical traits pointing to specialized biosynthetic capacity—exemplified by the prominence of bursehernin and α-tocopherol. These findings harmonize with Micevski’s [4] suggestion that *B. falcatum* var. *orbelicum* or var. *montenegrinum* could occur on Galičica, although the specific morphological criteria for distinguishing these varieties are not thoroughly documented. Nonetheless, the narrower, petiolate basal leaves in the Galičica sample compared to other taxa were noted, further highlighting the need for targeted morphological and molecular analyses.

Similarly, the sample from Mt. Olympus, characterized by linear and sessile basal leaves, has been assigned to *B. falcatum* subsp. *cernuum* (*syn*. *B. sibthorpianum*), consistent with Nikolić’s [3] distribution records for that taxon in the region. The distinctive phytochemical profile of its BS extract included 10-nonacosanone at exceptionally high levels and stereoisomeric constituents—a stereoisomer of compound **1** (6.1%) and two stereoisomers (4.9% and 5.2%) of compound **15**—reinforces its distinct status. Such metabolic divergence appears consistent with the morphologically well-separated *B. sibthorpianum*. The high abundance of 10-nonacosanone, the presence of *n*-alkanes, and newly detected polyunsaturated esters underscore significant metabolic divergence among populations of *B. falcatum sensu lato*. These findings lend further support to the hypothesis that morphological differences—particularly leaf shape, petiole length, and overall habit—are accompanied by distinct secondary metabolite profiles that may serve as taxonomic markers. For instance, the predominance of 10-nonacosanone in the Suva planina and Galičica samples contrasts with the more diverse polyunsaturated metabolite repertoire observed in the Šar planina population, suggesting divergent evolutionary histories or adaptations to microclimatic conditions.

Collectively, these results underscore the importance of integrating morphological, phytochemical, and molecular approaches to resolve the taxonomic uncertainties within the *Bupleurum falcatum* complex. The presence of multiple chemotypes—often mirroring nuanced morphological traits—attests to the evolutionary and ecological plasticity within these taxa. Future studies focusing on morphological, cytological, and molecular aspects, followed by metabolite profiling, are warranted to clarify the phylogenetic relationships among *B. falcatum sensu lato*, *B. sibthorpianum*, and their allied taxa in the Balkans.

## 3. Materials and Methods

### 3.1. General Experimental Procedures

All the chemicals and solvents used in this study were obtained from commercial suppliers (Sigma-Aldrich, St. Louis, MO, USA; Merck, Darmstadt, Germany; Fisher Scientific, Waltham, MA, USA) and utilized without further purification, except for the solvents, which were pre-distilled and dried prior to use. For preparative separations, including both dry-flash and column chromatography, silica gel 60 (0.04–0.063 mm, Merck, Darmstadt, Germany) was employed. Tung oil, marketed under the trade name Belinka Oil Tung, was procured from Helios Srbija a.d. (Gornji Milanovac, Serbia).

NMR spectra (^1^H and ^13^C) were recorded on a Bruker Avance III 400 MHz spectrometer (Fällanden, Switzerland), operating at 400 MHz for ^1^H and 100.6 MHz for ^13^C, equipped with a 5 mm dual ^13^C/^1^H probe at 20 °C. The spectra were acquired in chloroform-*d*, methanol-*d*_4_, or DMSO-*d*_6_ (Sigma-Aldrich, St. Louis, MO, USA), with tetramethylsilane (TMS) serving as the internal standard. Chemical shifts (*δ*) are expressed in ppm and referenced to TMS (*δ*_H_ = 0.00 ppm) or residual solvent peaks (CHCl_3_ for ^1^H; ^13^CDCl_3_, for ^13^C). Scalar coupling constants (*J*) are given in Hertz (Hz). Standard Bruker pulse sequences were used for acquiring both 1D and 2D NMR spectra.

GC-MS analyses (three replicates) were performed on a Hewlett-Packard 6890N gas chromatograph, fitted with a DB-5MS fused silica capillary column (5% polydiphenyl-siloxane and 95% polydimethylsiloxane, 30 m × 0.25 mm, 0.25 µm film thickness, Agilent Technologies, Santa Clara, CA, USA) and coupled to a 5975B mass selective detector from the same manufacturer. Retention indices (RIs) were determined using a series of *n*-alkanes (C_8_–C_40_). An HRMS analysis was conducted on a JEOL MStation JMS-700 mass spectrometer (EI ionization). UV measurements were carried out on a Shimadzu UV-1800 UV–vis spectrophotometer (Kyoto, Japan).

Analytical TLC was conducted on silica-gel-coated aluminum plates (Kieselgel 60 F_254_, 0.2 mm, Merck, Darmstadt, Germany). TLC visualization was initially performed under UV light (254 nm), followed by spraying with 50% (*w*/*w*) aqueous H_2_SO_4_ and heating.

The full ^1^H NMR spin analysis for compounds **1**, **2**, and **8** was conducted by manually refining *δ*_H_ and *J* values to match the experimentally obtained data, followed by further optimization using MestReNova 11.0.3 software (tools/spin simulation). This approach enabled a systematic adjustment of all the calculated NMR parameters, ultimately achieving excellent agreement between the simulated and experimental data (NRMSD < 0.05%) for the isolated compounds.

### 3.2. Plant Material

Three samples of the above-ground plant parts of *B. falcatum* subsp. *falcatum* (*B. falcatum*) were collected during flowering time from the slopes of Šar Planina Mt. (Kosovo, Serbia, BFK, 20 August 2022), Suva Planina Mt. (Serbia, BFS, 27 August 2023), and Galičica Mt. (North Macedonia, BFG, 8 August 2023). Furthermore, one sample of the aerial parts of *B. falcatum* subsp. *cernuum* (*B. sibthorpianum*) was collected on 10 August 2022 on Olympus Mt. (Greece, BS), all from single populations. Voucher specimens have been deposited in the Herbarium Moesiacum Niš (HMN), Faculty of Sciences and Mathematics, University of Niš (voucher nos. HMN-18290—BFK; HMN-18293—BFS; HMN-18292—BFG; and HMN-16265—BS). The identification of the material was conducted according to the *Flora of the Socialist Republic of Serbia* [3], *Flora of the Republic of Macedonia* [4], and *Flora Europaea* [5] and was performed by one of the co-authors (I. Lj. R.), who holds a PhD in botany.

### 3.3. Extraction and Isolation

The aerial parts of all plant samples were cut into small fragments and individually extracted through maceration with diethyl ether (Et_2_O) over a period of ten days, with occasional agitation at room temperature in the dark. After extraction, the extracts were dried using anhydrous magnesium sulfate (MgSO_4_) and then gravity filtered to eliminate insoluble material. The diethyl ether was subsequently removed under reduced pressure at room temperature. The yields of the extracts were 2.1%, 2.9%, 2.0%, and 1.8% (*w*/*w*, based on plant mass), for BFS, BFK, BFG, and BS, respectively. The BFS extract was subjected to silica gel dry-flash chromatography under gradient conditions using mixtures of increasing polarity, starting with hexane–Et_2_O, through Et_2_O-EtOAc, and ending in EtOAc–methanol mixtures. The chromatography yielded 30 fractions, which were pooled based on TLC and/or GC-MS analyses. Fraction 10 (eluted with hexane–Et_2_O 12:1, *v*/*v*) primarily contained long-chain aldehydes and methyl esters of fatty acids. Fraction 11 (hexane–Et_2_O 6:1, *v*/*v*) contained compounds **1**, **2**, 10-nonacosanone, and minor components. Fraction 13 (hexane–Et_2_O 4:1, *v*/*v*) comprised acetates of alcohols **13**–**16** (corresponding to compounds **17**–**20**). From fraction 18, compound **6** was identified. Fraction 19 (hexane–Et_2_O 3:2, *v*/*v*) consisted of a mixture of compounds **13** and **14** (pentadecatrienediyn-1-ols), together with bupleurynol and its stereoisomer (compounds **15** and **16**). Fraction 20 (hexane–Et_2_O 1:4, *v*/*v*) was pure compound **13** ((2*E*,8*E*,10*E*)-pentadecatriene-4,6-diyn-1-ol). Fraction 23 (eluted with pure diethyl ether) contained compound **7** as part of a complex mixture. Fraction 26 (eluted with pure ethyl acetate) was pure compound **21**. Fraction 11 was subjected to further separation by silica gel column chromatography. The column was packed with silica gel 60 and pre-equilibrated with *n*-hexane. Elution was performed under gradient conditions using mixtures of *n*-hexane and Et_2_O of increasing polarity. Fractions of 10 mL were collected, and the fractions showing similar TLC profiles were pooled. One of the pooled fractions containing compounds **1** and **2** was further purified by column chromatography on silica gel impregnated with 10% (*w*/*w*) silver nitrate under gradient conditions. The silver nitrate-impregnated silica gel was prepared by mixing silica gel with a 10% (*w*/*w*) acetonitrile solution of AgNO_3_, followed by drying at 50 °C overnight under reduced pressure. This step yielded a fraction enriched in compound **2**.

The BFG extract was subjected to silica gel dry-flash chromatography under the same conditions as described for the BFS extract.

### 3.4. Synthesis of Compounds ***6*** and ***17***–***20***

Two separate acetylation reactions were conducted: one involving fraction 19, leading to the formation of compounds **17**–**20**, and the other involving fraction 23, resulting in the formation of compound **6**. Thus, solutions of fraction 19 or fraction 23 (approx. 10 mg), acetic acid (5 mg), 4-(dimethylamino)pyridine (DMAP, 4 mg), and *N,N′*-dicyclohexylcarbodiimide (DCC, 25 mg) in 1 mL of dry CH_2_Cl_2_ were stirred overnight at room temperature under an argon atmosphere. Afterward, the solvent was removed in vacuo; then, 3 mL of cold pentane was added to the residue, and the precipitated *N*,*N*′-dicyclohexylurea was filtered off. The filtrate was concentrated in vacuo, and directly analyzed by GC-MS.

### 3.5. Reduction of Tung Oil

A solution of tung oil (2 g) in anhydrous tetrahydrofuran (THF, 20 mL) was slowly added in portions to a previously prepared suspension of lithium aluminum hydride (0.03 mol, 1.15 g) in dry THF (20 mL) over a 30 min period. The reaction mixture was then stirred for an additional hour under magnetic stirring. Subsequently, 100 mL of 10% NaOH (aq, *w*/*w*) solution was added to the reaction mixture. The THF solvent was removed under reduced pressure, and the resulting residue was extracted exhaustively three times with diethyl ether. The organic layers were combined, dried over anhydrous MgSO_4_, and the solvent was removed under reduced pressure. The resulting mixture (1.55 g) was analyzed by GC-MS and was found to be primarily composed of the reduction product (9*Z*,11*E*,13*E*)-octadeca-9,11,13-trien-1-ol (**4**).

### 3.6. Synthesis of Compound ***5***

A solution of reduced tung oil (530 mg), tiglic acid ((*E*)-2-methylbut-2-enoic acid, 200 mg, 2 mmol), 4-(dimethylamino)pyridine (DMAP, 24 mg, 0.2 mmol), and *N*,*N*′-dicyclohexylcarbodiimide (DCC, 512 mg, 2 mmol) in 20 mL of dry CH_2_Cl_2_ was stirred in a round-bottomed flask overnight at room temperature, under argon. Afterward, the solvent was removed in vacuo; then, 10 mL of cold pentane was added to the residue, and the precipitated *N*,*N*′-dicyclohexylurea was filtered off. The filtrate was concentrated in vacuo, and the resulting residue was purified by silica gel column chromatography giving 484 mg (a 70% yield) of (9*Z*,11*E*,13*E*)-octadeca-9,11,13-trien-1-yl tiglate.

### 3.7. Experimental Spectral Data

(5*Z*,7*E*,9*E*,11*Z*)-tetradeca-5,7,9,11-tetraen-1-yl (*E*)-2-methylbut-2-enoate (**1**): retention index (RI) = 2331 (DB-5MS column); UV (CH_3_CN) λ_max_(log ε) 237 (4.17), 289 (4.25), 302 (4.55), 316 (4.49), 341 (2.95) nm; ^1^H-NMR (400 MHz) and ^13^C-NMR (100.6 MHz) in CDCl_3_ are given in Table 1 (see Section 2); MS (EI), *m*/*z* (%): 288 (40), 131 (54), 117 (63), 105 (41), 91 (89), 83 (95), 79 (44), 67 (31), 55 (100), 41 (30); HRMS (EI) calc. for C_19_H_28_O_2_ [M]^+^: 288.2089, found: 288.2094.

(5*Z*,7*E*,9*E*)-tetradeca-5,7,9-trien-1-yl (*E*)-2-methylbut-2-enoate (**2**): retention index (RI) = 2253 (DB-5MS column); the UV of an enriched fraction with **2** (CH_3_CN) λ_max_(log ε) 236 (4.15), 270 (2.15) nm; ^1^H-NMR (400 MHz) and ^13^C-NMR (100.6 MHz) in CDCl_3_ are given in Table 1 (see Section 2); MS (EI), *m*/*z* (%): 290 (21), 119 (25), 105 (37), 93 (21), 91 (58), 80 (21), 79 (34), 77 (20), 83 (100), 55 (51); HRMS (EI) calc. for C_19_H_30_O_2_ [M]^+^: 290.2246, found: 290.2251.

(9*Z*,11*E*,13*E*)-octadeca-9,11,13-trien-1-ol (**4**): retention index (RI) = 2214 (DB-5MS column); MS (EI), *m*/*z* (%): 264 (47), 105 (29), 93 (71), 91 (100), 80 (47), 79 (86), 77 (48), 67 (39), 55 (33), 41 (44). The stereochemistry is assumed based on the stereochemistry of the starting material and the product.

(9*Z*,11*E*,13*E*)-octadeca-9,11,13-trien-1-yl (*E*)-2-methylbut-2-enoate (**5**): retention index (RI) = 2652 (DB-5MS column); ^1^H-NMR (400 MHz) and ^13^C-NMR (100.6 MHz) in CDCl_3_ are given in the Supplementary Material; MS (EI), *m*/*z* (%): 346 (40), 105 (43), 101 (61), 93 (72), 91 (95), 83 (86), 80 (62), 79 (100), 67 (45), 55 (92).

(2*E*,8*E*,10*E*)-14-oxoheptadeca-2,8,10-trien-4,6-diyn-1-yl acetate (**6**): retention index (RI) = 2730 (DB-5MS column); ^1^H NMR (400 MHz, CDCl_3_): *δ* 6.67 (dd, *J* = 15.4, 11.0 Hz, 1 H, H-9′), 6.28 (dt, *J* = 15.9, 5.8 Hz, 1 H, H-2′), 6.12 (dddt, *J* = 15.0, 10.7, 1.4, 0.7 Hz, 1 H, H-10′), 5.87–5.80 (m, 2 H, H-3′ and H-11′), 5.56 (d, *J* = 15.6 Hz, 1 H, H-8′), 4.62 (dd, *J* = 5.8, 1.7 Hz, 2 H, CH_2_, H-1′), 2.51 (t, *J* = 7.1 Hz, 2 H, CH_2_, H-13′), 2.41–2.35 (m, 4 H, CH_2_, H-12′ and H-15′), 2.08 (s, 3 H, CH_3_, H-2), 1.62–1.55 (m, 2 H, CH_2_, H-16′), 0.91 (t, *J* = 7.4 Hz, 3 H, CH_3_, H-17′); ^13^C NMR (100.6 MHz, CDCl_3_): *δ* 209.7 (C-4′), 170.6 (C-1), 145.2 (C-9′), 139.6 (C-2′), 138.1 (C-11′), 130.3 (C-10′), 112.4 (C-3′), 108.1 (C-8′), 81.8 (C-7′), 79.8 (C-4′), 75.8 (C-6′), 75.7 (C-5′), 63.8 (C-1′), 45.0 (C-12′), 41.8 (C-13′), 27.0 (C-15′), 20.9 (C-2), 17.4 (C-16′), 13.8 (C-17′). The NMR spectral data are in complete agreement with the values published by Sommerwerk et al., 2015 [19]; MS (EI), *m*/*z* (%): 298 (2), 169 (25), 167 (31), 165 (23), 153 (21), 152 (38), 141 (26), 115 (26), 71 (45), 43 (100); HRMS (EI) calc. for C_19_H_22_O_3_ [M]^+^: 298.1569, found: 298.1574.

(7*E*,9*E*,15*E*)-17-hydroxyheptadeca-7,9,15-trien-11,13-diyn-4-one (**7**): retention index (RI) = 2618 (DB-5MS column); MS (EI), *m*/*z* (%): 256 (6), 167 (46), 152 (48), 141 (81), 129 (36), 128 (64), 127 (36), 115 (80), 71 (56), 43 (100).

(8*E*,10*E*)-heptadeca-8,10-dien-2,4,6-triyne (**8**): retention index (RI) = 2210 (DB-5MS column); UV (CH_3_CN) λ_max_(log ε) 258 (4.11), 268 (4.48), 304 (4.26) nm; ^1^H-NMR (400 MHz) and ^13^C-NMR (100.6 MHz) in CDCl_3_ are given in Table 2 (see Section 2); MS (EI), *m*/*z* (%): 224 (53), 165 (46), 153 (94), 152 (100), 151 (29), 140 (60), 139 (68), 127 (26), 115 (27), 41 (21); HRMS (EI) calc. for C_17_H_20_ [M]^+^: 224.1565, found: 224.1570.

(2*E*,8*E*,10*E*)-pentadeca-2,8,10-trien-4,6-diyn-1-ol (**13**): retention index (RI) = 2196 (DB-5MS column); MS (EI), *m*/*z* (%): 214 (51), 141 (33), 129 (62), 128 (100), 127 (51), 116 (21), 115 (84), 91 (26), 77 (28), 41 (23).

(2*Z*,8*E*,10*E*)-pentadeca-2,8,10-trien-4,6-diyn-1-ol (**14**): retention index (RI) = 2116 (DB-5MS column); MS (EI), *m*/*z* (%): 214 (43), 152 (19), 141 (27), 129 (57), 128 (100), 127 (40), 115 (72), 91 (28), 77 (23), 41 (19).

(2*E*,8*E*,10*E*)-heptadeca-2,8,10-trien-4,6-diyn-1-ol (oenanthetol, **15**): retention index (RI) = 2416 (DB-5MS column); MS (EI), *m*/*z* (%): 242 (70), 141 (37), 129 (81), 128 (100), 127 (51), 116 (28), 115 (86), 91 (27), 77 (27), 41 (25).

(2*Z*,8*E*,10*E*)-heptadeca-2,8,10-trien-4,6-diyn-1-ol (bupleurynol, **16**): retention index (RI) = 2338 (DB-5MS column); MS (EI), *m*/*z* (%): 242 (44), 157 (27), 141 (30), 129 (71), 128 (100), 127 (39), 115 (79), 91 (28), 77 (24), 41 (24).

(2*E*,8*E*,10*E*)-pentadeca-2,8,10-trien-4,6-diyn-1-yl acetate (**17**): retention index (RI) = 2310 (DB-5MS column); MS (EI), *m*/*z* (%): 256 (80), 157 (32), 153 (41), 152 (42), 141 (31), 129 (43), 128 (65), 127 (31), 115 (51), 43 (100).

(2*Z*,8*E*,10*E*)-pentadeca-2,8,10-trien-4,6-diyn-1-yl acetate (**18**): retention index (RI) = 2245 (DB-5MS column); MS (EI), *m*/*z* (%): 256 (67), 157 (29), 153 (34), 152 (35), 141 (30), 129 (41), 128 (65), 127 (28), 115 (50), 43 (100).

(2*E*,8*E*,10*E*)-heptadeca-2,8,10-trien-4,6-diyn-1-yl acetate (**19**): retention index (RI) = 2532 (DB-5MS column); MS (EI), *m*/*z* (%): 284 (50), 241 (22), 157 (30), 153 (29), 152 (28), 141 (23), 129 (30), 128 (43), 115 (36), 43 (100).

(2*Z*,8*E*,10*E*)-heptadeca-2,8,10-trien-4,6-diyn-1-yl acetate (**20**): retention index (RI) = 2463 (DB-5MS column); MS (EI), *m*/*z* (%): 284 (22), 157 (17), 153 (15), 152 (15), 141 (114), 129 (18), 128 (28), 115 (26), 41 (17), 43 (100).

9-(3,4-dimethoxyphenyl)-5,9-dihydro-8*H*-furo[3′,4′:6,7]naphtho[2,3-*d*][1,3]dioxol-6-one (**21**): retention index (RI) = 3357 (DB-5MS column); MS (EI), *m*/*z* (%): 367 (22), 366 (100), 321 (22), 291 (18), 290 (13), 199 (14), 185 (34), 165 (11), 139 (11), 138 (62).

Compound **17** stereoisomer: retention index (RI) = 2219 (DB-5MS column); MS (EI), *m*/*z* (%): 256 (10), 213 (76), 157 (50), 153 (57), 152 (51), 129 (51), 128 (77), 141 (40), 115 (63), 43 (100).

Praealtaester B: (RI) = 2229 (DB-5MS column); MS (EI), *m*/*z* (%): 290 (56), 131 (57), 117 (66), 91 (70), 85 (45), 79 (41), 67 (53), 57 (100), 55 (36), 41 (45).

Praealtaester B stereoisomer 1: retention index (RI) = 2232 (DB-5MS column); MS (EI), *m*/*z* (%): 290 (47), 145 (59), 131 (66), 119 (47), 117 (55), 105 (55), 91 (100), 85 (68), 79 (53), 41 (33).

Praealtaester B stereoisomer 2: retention index (RI) = 2236 (DB-5MS column); MS (EI), *m*/*z* (%): 290 (77), 159 (37), 145 (40), 131 (65), 117 (67), 105 (46), 91 (100), 79 (46), 57 (97), 41 (45).

Praealtaester B stereoisomer 3: retention index (RI) = 2240 (DB-5MS column); MS (EI), *m*/*z* (%): 290 (86), 145 (40), 131 (61), 117 (69), 105 (48), 91 (100), 85 (56), 79 (50), 57 (52), 41 (45).

Praealtaester B stereoisomer 4: retention index (RI) = 2270 (DB-5MS column); MS (EI), *m*/*z* (%): 290 (88), 159 (40), 131 (64), 117 (73), 105 (42), 91 (100), 85 (41), 79 (45), 57 (94), 41 (47).

Praealtaester B stereoisomer 5: retention index (RI) = 2274 (DB-5MS column); MS (EI), *m*/*z* (%): 290 (85), 145 (36), 131 (55), 117 (67), 105 (46), 91 (100), 85 (59), 79 (45), 57 (45), 41 (43).

Compound **1** stereoisomer 1: retention index (RI) = 2341 (DB-5MS column); MS (EI), *m*/*z* (%): 288 (59), 145 (29), 131 (54), 117 (57), 105 (36), 91 (76), 83 (100), 79 (39), 67 (26), 55 (69).

Compound **15** stereoisomer 1: retention index (RI) = 2358 (DB-5MS column); MS (EI), *m*/*z* (%): 242 (64), 157 (34), 141 (42), 129 (74), 128 (100), 127 (38), 115 (75), 91 (31), 77 (23), 43 (23).

Compound **1** stereoisomer 2: retention index (RI) = 2371 (DB-5MS column); MS (EI), *m*/*z* (%): 288 (47), 159 (20), 145 (25), 131 (45), 117 (52), 91 (68), 83 (100), 79 (34), 67 (21), 55 (57).

Compound **1** stereoisomer 3: retention index (RI) = 2378 (DB-5MS column); MS (EI), *m*/*z* (%): 288 (60), 145 (28), 131 (52), 117 (55), 105 (34), 91 (74), 83 (100), 79 (38), 67 (25), 55 (69).

Compound **15** stereoisomer 2: retention index (RI) = 2434 (DB-5MS column); MS (EI), *m*/*z* (%):242 (93), 157 (26), 141 (36), 129 (87), 128 (100), 127 (54), 116 (29), 115 (86), 91 (30), 77 (26).

## 4. Conclusions

This study revealed a suite of newly discovered polyunsaturated esters and related less-polar metabolites in *Bupleurum falcatum sensu lato* and *B. sibthorpianum* across four Balkan populations. While earlier studies of *Bupleurum* species often focused on polar saponins, this work highlights the chemical richness of less-polar fractions and their potential taxonomic utility. Notably, several polyacetylenes diverged from classic falcarinol-type scaffolds, reflecting a possible offshoot of the crepenynate pathway. Differences in the metabolite profiles among *B. falcatum* subsp. *falcatum*, *B. falcatum* f. *latifolium*, and *B. falcatum* subsp. *cernuum* (*syn*. *B. sibthorpianum*) paralleled morphological distinctions, suggesting these new compounds may serve as chemotaxonomic markers. By characterizing these novel metabolites, new avenues were opened for research into their biosynthesis, bioactivity, and evolutionary significance. A multidisciplinary approach that integrates detailed morphological, cytological, molecular, and phylogenetic analyses, alongside biological testing, will be essential in further clarifying the relationships and potential medicinal value within this taxonomically complex *Bupleurum falcatum sensu lato* group.

## Figures and Tables

**Table 1:** The ^1^H (400 MHz) and ^13^C (100.6 MHz) NMR spectral data (in CDCl_3_) of compounds **1** and **2** (NMR parameters are derived from manual iterative full spin analyses).

	Compound 1		Compound 2		
Position	*δ*_H_ (m, *J* ^1^ (Hz), Integral)	*δ*_C_ (ppm)	Position	*δ*_H_ (m, *J* (Hz), Integral)	*δ*_C_ (ppm)
1	/	168.32	1	/	168.33
2	/	128.87	2	/	128.85
3	6.85 (qq, ^3^*J*_3,4_ = 7.1, ^4^*J*_3,5_ = 1.4, 1 H)	137.07	3	6.85 (qq, ^3^*J*_3,4_ = 7.0, ^4^*J*_3,5_ = 1.5, 1 H)	137.30
4	1.78 (d, ^3^*J*_3,4_ = 7.1, 3 H)	14.45	4	1.79 (dq, ^3^*J*_3,4_ = 7.0, ^5^*J*_4,5_ = 1.2, 3 H)	14.39
5	1.83 (d, ^4^*J*_3,5_ = 1.4, 3 H)	12.17	5	1.83 (dq, ^4^*J*_3,5_ = 1.5, ^5^*J*_4,5_ = 1.2, 3 H)	11.78
1′a	4.13 (dd, ^3^*J*_1′a,2′b_ = 7.7, ^3^*J*_1′a,2′a_ = 5.5, 1 H)	64.39	1′a	4.13 (dd, ^3^*J*_1′a,2′b_ = ^3^*J*_1′a,2′a_ = 7.0, 1 H)	64.16
1′b	4.13 (dd, ^3^*J*_1′b,2′a_ = 7.7, ^3^*J*_1′b,2′b_ = 5.5, 1 H)		1′b	4.13 (dd, ^3^*J*_1′b,2′a_ = ^3^*J*_1′b,2′b_ = 7.0, 1 H)	
2′a	1.69 (dddd, ^3^*J*_2′a,3′b_ = 9.8, ^3^*J*_1′b,2′a_ = 7.7, ^3^*J*_2′a,3′a_ = 5.6, ^3^*J*_1′a,2′a_ = 5.5, 1 H)	28.44	2′a	1.69 (dddd, ^3^*J*_2′a,3′b_ = 9.0, ^3^*J*_1′b,2′a_ = ^3^*J*_1′a,2′a_ = 7.0, ^3^*J*_2′a,3′a_ = 5.0, 1 H)	26.15
2′b	1.69 (dddd, ^3^*J*_2′b,3′a_ = 9.8, ^3^*J*_1′a,2′b_ = 7.7, ^3^*J*_2′b,3′b_ = 5.6, ^3^*J*_1′b,2′b_ = 5.5, 1 H)		2′b	1.69 (dddd, ^3^*J*_2′b,3′a_ = 9.0, ^3^*J*_1′a,2′b_ = ^3^*J*_1′b,2′b_ = 7.0, ^3^*J*_2′b,3′b_ = 5.0, 1 H)	
3′a	1.49 (dddd, ^3^*J*_2′b,3′a_ = ^3^*J*_3′a,4′b_ = 9.8, ^3^*J*_2′a,3′a_ = ^3^*J*_3′a,4′a_ = 5.6, 1 H)	26.23	3′a	1.48 (dddd, ^3^*J*_2′b,3′a_ = ^3^*J*_3′a,4′b_ = 9.0, ^3^*J*_2′a,3′a_ = ^3^*J*_3′a,4′a_ = 5.0, 1 H)	26.95
3′b	1.49 (dddd, ^3^*J*_2′a,3′b_ = ^3^*J*_3′b,4′a_ = 9.8, ^3^*J*_2′b,3′b_ = ^3^*J*_3′b,4′b_ = 5.6, 1 H)		3′b	1.48 (dddd, ^3^*J*_2′a,3′b_ = ^3^*J*_3′b,4′a_ = 9.0, ^3^*J*_2′b,3′b_ = ^3^*J*_3′b,4′b_ = 5.0, 1 H)	
4′a	2.23 (dddd, ^3^*J*_3′b,4′a_ = 9.8, ^3^*J*_4′a,5′_ = 7.8, ^3^*J*_3′a,4′a_ = 5.6, ^4^*J*_4′a,6′_ = 1.7, 1 H)	27.61	4′a	2.12 (dddd, ^3^*J*_3′b,4′a_ = 9.0, ^3^*J*_4′a,5′_ = 7.3, ^3^*J*_3′a,4′a_ = 5.0, ^4^*J*_4′a,6′_ = 1.3, 1 H)	27.58
4′b	2.23 (dddd, ^3^*J*_3′a,4′b_ = 9.8, ^3^*J*_4′b,5′_ = 7.8, ^3^*J*_3′b,4′b_ = 5.6, ^4^*J*_4′b,6′_ = 1.7, 1 H)		4′b	2.12 (dddd, ^3^*J*_3′a,4′b_ = 9.0, ^3^*J*_4′b,5′_ = 7.3, ^3^*J*_3′b,4′b_ = 5.0, ^4^*J*_4′b,6′_ = 1.3, 1 H)	
5′	5.46 (dddd, ^3^*J*_5′,6′_ = 11.0, ^3^*J*_4′a,5′_ = ^3^*J*_4′b,5′_ = 7.8, ^4^*J*_5′,7′_ = −1.7, 1 H)	134.76	5′	5.38 (dddd, ^3^*J*_5′,6′_ = 10.8, ^3^*J*_4′a,5′_ = ^3^*J*_4′b,5′_ = 7.3, ^4^*J*_5′,7′_ = −0.8, 1 H)	132.03
6′	6.06 (ddddd, ^3^*J*_5′,6′_ = ^3^*J*_6′,7′_ = 11.0, ^4^*J*_4′a,6′_ = ^4^*J*_4′b,6′_ = 1.7, ^4^*J*_6′,8′_ = −0.7, 1 H)	129.39	6′	6.02 (ddddd, ^3^*J*_6′,7′_ = 11.4, ^3^*J*_5′,6′_ = 10.8, ^4^*J*_4′a,6′_ = ^4^*J*_4′b,6′_ = 1.3, ^4^*J*_6′,8′_ = −0.7, 1 H)	129.34
7′	6.50 (ddddd, ^3^*J*_7′,8′_ = 15.2, ^3^*J*_6′,7′_ = 11.0, ^4^*J*_5′,7′_ = −1.7, ^4^*J*_7′,9′_ = −0.8, ^5^*J*_7′,10′_ = −0.6, 1 H)	128.40	7′	6.36 (dddd, ^3^*J*_7′,8′_ = 14.8, ^3^*J*_6′,7′_ = 11.4, ^4^*J*_5′,7′_ = −0.8, ^4^*J*_7′,9′_ = −0.7, 1 H)	125.86
8′	6.27 (dddd, ^3^*J*_7′,8′_ = 15.2, ^3^*J*_8′,9′_ = 12.0, ^4^*J*_8′,10′_ = −0.8, ^4^*J*_6′,8′_ = −0.7, 1 H)	133.27	8′	6.17 (dddd, ^3^*J*_7′,8′_ = 14.8, ^3^*J*_8′,9′_ = 10.8, ^4^*J*_8′,10′_ = −0.7, ^4^*J*_6′,8′_ = −0.7, 1 H)	132.03
9′	6.26 (dddd, ^3^*J*_9′,10′_ = 15.2, ^3^*J*_8′,9′_ = 12.0, ^4^*J*_7′,9′_ = −0.8, ^4^*J*_9′,11′_ = −0.7, 1 H)	132.83	9′	6.0980 (ddddd, ^3^*J*_9′,10′_ = 15.0, ^3^*J*_8′,9′_ = 10.8, ^4^*J*_7′,9′_ = −0.7, ^4^*J*_9′,11′a_ = ^4^*J*_9′,11′b_ = 1.3, 1 H)	130.62
10′	6.48 (ddddd, ^3^*J*_9′,10′_ = 15.2, ^3^*J*_10′,11′_ = 11.0, ^4^*J*_10′,12′_ = 1.0, ^4^*J*_8′,10′_ = −0.8, ^5^*J*_7′,10′_ = −0.6, 1 H)	128.04	10′	5.71 (dddd, ^3^*J*_9′,10′_ = 15.0, ^3^*J*_10′,11′a_ = ^3^*J*_10′,11′b_ = 6.9, ^4^*J*_8′,10′_ = −0.7, 1 H)	135.60
11′	6.01 (ddddd, ^3^*J*_10′,11′_ = ^3^*J*_11′,12′_ = 11.0, ^4^*J*_11′,13′a_ = ^4^*J*_11′,13′b_ = 1.6, ^4^*J*_9′,11′_ = −0.7, 1 H)	128.25	11′a	2.10 (dddd, ^3^*J*_10′,11′a_ = 6.9, ^3^*J*_11′a,12′a_ = ^3^*J*_11′a,12′b_ = 7.2, ^4^*J*_9′,11′a_ = 1.3, 1 H)	32.64
			11′b	2.10 (dddd, ^3^*J*_10′,11′b_ = 6.9, ^3 3^*J*_11′b,12′a_ = ^3^*J*_11′b,12′b_ = 7.2, ^4^*J*_9′,11′b_ = 1.3, 1 H)	
12′	5.43 (dddd, ^3^*J*_11′,12′_ = 11.0, ^3^*J*_12′,13′a_ = ^3^*J*_12′,13′b_ = 7.8, ^4^*J*_10′,12′_ = 1.0, 1 H)	132.10	12′a	1.29 (dddd, ^3^*J*_12a′,13′b_ = 9.0, ^3^*J*_11′a,12′a_ = ^3^*J*_11′b,12′a_ = 7.2, ^3^*J*_12a′,13′a_ = 5.0, 1 H)	31.58
			12′b	1.29 (dddd, ^3^*J*_12b′,13′a_ = 9.0, ^3^*J*_11′a,12′b_ = ^3^*J*_11′b,12′b_ = 7.2, ^3^*J*_12b′,13′b_ = 5.0, 1 H)	
13′a	2.24 (ddd, ^3^*J*_12′,13′a_ = 7.8, ^3^*J*_13′a,14′_ = 7.5, ^4^*J*_11′,13′a_ = 1.6, 1 H)	21.39	13′a	1.37 (ddd, ^3^*J*_12b′,13′a_ = 9.0, ^3^*J*_13′a, 14′_ = 7.0, ^3^*J*_12a′,13′a_ = 5.0, 1 H)	22.55
13′b	2.24 (ddd, ^3^*J*_12′,13′b_ = 7.8, ^3^*J*_13′b,14′_ = 7.5, ^4^*J*_11′,13′b_ = 1.6, 1 H)		13′b	1.37 (ddd, ^3^*J*_12a′,13′b_ = 9.0, ^3^*J*_13′b,14′_ = 7.0, ^3^*J*_12b′,13′b_ = 5.0, 1 H)	
14′	1.01 (t, ^3^*J*_13′a, 14′_ = ^3^*J*_13′b,14′_ = 7.5, 3 H)	14.38	14′	0.90 (t, ^3^*J*_13′a, 14′_ = ^3^*J*_13′b,14′_ = 7.0, 3 H)	14.07

^1^ The coupling constant values were initially inferred from the ^1^H homoselective decoupling NMR experiments and afterward refined through a manual iterative full spin analysis. For details, see Section 3.

**Table 2:** The ^1^H (400 MHz) and ^13^C (100.6 MHz) NMR spectral data (in CDCl_3_) of compound **8** (NMR parameters are derived from manual iterative full spin analyses).

Position	*δ*_H_ (m, *J* ^1^ (Hz), Integral)	*δ*_C_ (ppm)
1	1.99 (s, 3 H)	4.86
2	/ ^2^	78.55
3	/	65.19
4	/	67.99
5	/	59.50
6	/	76.46
7	/	75.62
8	5.48 (ddd, ^3^*J*_8,9_ = 15.6, ^4^*J*_8,10_ = 0.7, ^5^*J*_8,11_ = 0.6, 1 H)	106.75
9	6.74 (ddd, ^3^*J*_8,9_ = 15.6, ^3^*J*_9,10_ = 10.9, ^4^*J*_9,11_ = 0.6, 1 H)	147.02
10	6.10 (ddtd, ^3^*J*_10,11_ = 15.1, ^3^*J*_9,10_ = 10.9, ^4^*J*_10,12_ = 1.3, ^4^*J*_8′,10′_ = 0.7, 1 H)	129.55
11	5.89 (dtdd, ^3^*J*_10,11_ = 15.1, ^3^*J*_11,12_ = 7.2, ^4^*J*_9,11_ = ^5^*J*_8′,11′_ = 0.6, 1 H)	141.07
12	2.12 (m, ^3^*J*_11,12_ = 7.2, ^4^*J*_10,12_ = 1.3, 2 H)	33.06
13 ^3^	1.37 (overlapped multiplets, 2 H)	28.98
14 ^3^	1.31 (overlapped multiplets, 2 H)	29.85
15 ^3^	1.25 (overlapped multiplets, 2 H)	31.79
16 ^3^	1.29 (m, ^3^*J*_16,17_ = 7.2, 2 H)	22.71
17	0.88 (t, ^3^*J*_16,17_ = 7.2, 3 H)	14.21

^1^ The coupling constant values were initially inferred from the ^1^H homoselective decoupling NMR experiments and afterward refined through a manual iterative full spin analysis. For details, see Section 3. ^2^ No proton in this position. ^3^ Severely overlapping multiplets; the signals were not simulated.

**Table 3:** Chemical composition of crude BFS, BFK, BFG, and BS extracts.

RI ^1^	RI ^2^	Compound ^3^	Relative Abundance ^4^			
			BFS	BFK	BFG	BS
1100	1100	Undecane ^5^	0.2	- ^6^	0.3	-
1424	1419	*β*-Ylangene	-	-	0.1	-
1420	1417	(*E*)-Caryophyllene ^5^	0.2	-	-	-
1489	1484	Germacrene D ^5^	4.1	-	tr ^7^	tr
1504	1500	Bicyclogermacrene	0.2	-	-	-
1512	1505	*β*-Bisabolene	-	-	0.1	-
1550	1542	(*E*)-*α*-Bisabolene	-	-	0.1	-
1844	1841	Neophytadiene (Isomer 1)	-	-	0.2	-
2116	/ ^8^	Compound **14**	2.2	4.5	-	-
2110	2104	(*E*)-Phytol ^5^	-	-	0.8	-
2196	/	Compound **13**	10.1	17.6	-	-
2210	/	Compound **8**	-	-	5.1	-
2219	/	Compound **17** stereoisomer ^9^	-	0.3	-	-
2229	/	Praealtaester B	0.6	-	-	-
2232	/	Praealtaester B stereoisomer 1	1.5	0.1	-	0.7
2236	/	Praealtaester B stereoisomer 2	-	0.3	-	0.7
2240	/	Praealtaester B stereoisomer 3	-	1.0	-	4.5
2245	/	Compound **18**	0.3	-	-	-
2253	/	Compound **2**	0.6	0.6	-	1.4
2270	/	Praealtaester B stereoisomer 4	-	0.6	-	1.6
2274	/	Praealtaester B stereoisomer 5	-	0.5	-	2.6
2300	2300	Tricosane ^5^	-	0.1	0.1	0.4
2310	/	Compound **17**	2.2	10.5	-	-
2331	/	Compound **1**	10.3	1.0	-	1.4
2338	/	Compound **16**	tr	0.7	1.8	-
2341	/	Compound **1** stereoisomer 1	3.4	4.7	-	6.1
2358	/	Compound **15** stereoisomer 1	-	-	-	4.9
2371	/	Compound **1** stereoisomer 2	0.3	1.2	-	1.3
2378	/	Compound **1** stereoisomer 3	tr	1.9	-	2.8
2416	/	Compound **15**	4.6	6.3	0.8	-
2432	2432	Docosanal ^5^	-	-	0.1	-
2434	/	Compound **15** stereoisomer 2	-	-	-	5.2
2500	2500	Pentacosane ^5^	0.4	0.1	0.5	0.8
2532	/	Compound **19**	0.5	4.8	0.1	-
2595	2595	1-Hexacosene	-	-	0.1	-
2600	2600	Hexacosane ^5^	-	-	0.1	-
2639	2632	Tetracosanal ^5^	0.2	0.4	0.2	0.4
2700	2700	Heptacosane ^5^	0.9	0.2	1.5	1.1
2742	2738	Pentacosanal	tr	0.1	-	tr
2800	2800	Octacosane ^5^	tr	-	-	-
2831	2835	(*E*,*E*,*E*,*E*)-Squalene ^5^	-	-	0.2	-
2845	2833	Hexacosanal ^5^	6.0	9.6	6.0	7.1
2900	2900	Nonacosane ^5^	-	0.3	1.1	1.0
2908	2906	1-Hexacosanol ^5^	9.8	-	3.0	0.9
2946	2944	Heptacosanal	0.3	0.1	0.3	-
3049	3040	Octacosanal ^5^	4.1	3.9	3.3	4.0
3097	3090	10-Nonacosanone	15.3	8.8	0.6	40.1
3100	3100	Hentriacontane ^5^	-	0.1	0.4	-
3111	3111	1-Octacosanol ^5^	6.7	4.8	-	-
3112	3111	10-Nonacosanol	-	-	9.5	-
3120	/	Bursehernin ^10^	-	-	21.3	-
3148	3149	*α*-Tocopherol ^5^	1.6	-	3.6	2.8
3175	/	Kaerophyllin ^11^	-	-	12.6	-
3186	/	Isokaerophyllin ^12^	-	-	2.7	-
3251	3250	Triacontanal	-	-	0.4	0.8
Total identified:			86.6	85.1	77.0	92.6

^1^ Retention indices determined experimentally on a DB-5MS column relative to a series of C_10_-C_40_ *n*-alkanes. ^2^ Values of retention indices from the literature, taken from Adams [31] or the NIST [32] collection, if not stated otherwise. ^3^ Compound identified based on mass spectra and retention indices matching with data from the literature. ^4^ Values are means of three individual analyses; the relative abundance (%) was determined by TIC chromatogram integration without the use of correction factors. ^5^ The constituent identity was confirmed by the co-injection of an authentic sample. ^6^ -, not detected in the analysis of crude extracts. ^7^ tr, trace amount (<0.05%). ^8^ not available; either a new compound or unknown. ^9^ Here, stereoisomer refers to diastereoisomers differing only in the configuration of double bonds. ^10^ MS (*m*/*z*): 371 (11), 370 (48), 177 (14), 152 (24), 151 (74), 136 (13), 135 (100), 107 (14), 105 (11), 77 (25) [33]. ^11^ MS (*m*/*z*): 368 (14), 234 (13), 233 (96), 177 (36), 146 (13), 135 (100), 131 (11), 115 (9), 77 (31), 51 (11) [34,35]. ^12^ MS (*m*/*z*): 368 (13), 234 (14), 233 (100), 177 (42), 146 (15), 135 (86), 131 (12), 115 (10), 77 (30), 51 (12) [34,35].

## Data Availability

The datasets and materials generated and/or analyzed during the current study are available from the corresponding author on reasonable request.

## Supplementary Materials

The following supporting information can be downloaded at https://www.mdpi.com/article/10.3390/plants14101432/s1. Figures S1–S28 and Tables S1–S3. Figures S1, S8 and S15: Key HMBC interactions; Figures S2, S3, S6, S7, S13 and S14: NMR spectra; Figures S5, S10, S12 and S17: HRMS spectra; Figures S4, S9, S11, S16 and S18–S28: Mass spectra; Tables S1–S3: NMR spectral data.
