# Supplementary material for: Unmasking the Aliphatic Repertoire: New Polyunsaturated Metabolites in Bupleurum falcatum sensu lato Provide Chemotaxonomic Insights
Source: Plants (Basel). 2025 May 10;14(10):1432. doi: 10.3390/plants14101432 (PMC12115290; doi:10.3390/plants14101432)
Supplement: Supplementary file 1 [file plants-14-01432-s001.zip › plants-3582666-supplementary.pdf]

Supplementary material

for

Unmasking the Aliphatic Repertoire: New Polyunsaturated Metabolites in *Bupleurum falcatum sensu lato* Provide Chemotaxonomic Insights

Milica D. Nešić [1], Milan S. Nešić [1], Irena Lj. Raca [2], Miha Bukleski [3] and Niko S. Radulović [1,*]

1 Department of Chemistry, Faculty of Sciences and Mathematics, University of Niš, Višegradska 33, 18000 Niš, Serbia; milica.stevanovic992@gmail.com (M.D.N.); milan.nesic@pmf.edu.rs (M.S.N.)
2 Department of Biology and Ecology, Faculty of Sciences and Mathematics, University of Niš, Višegradska 33, 18000 Niš, Serbia; irena.raca@pmf.edu.rs
3 Institute of Chemistry, Faculty of Natural Sciences and Mathematics, Ss. Cyril and Methodius University, Arhimedova 5, 1000 Skopje, North Macedonia; mihabukleski@yahoo.com
* Correspondence: nikoradulovic@yahoo.com

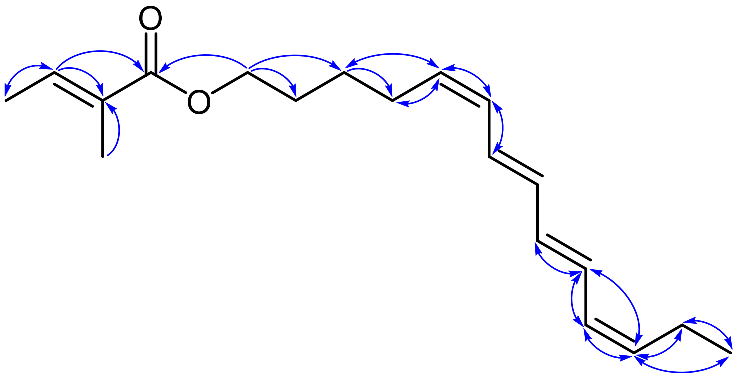

Figure S1. Important HMBC interactions of compound **1**

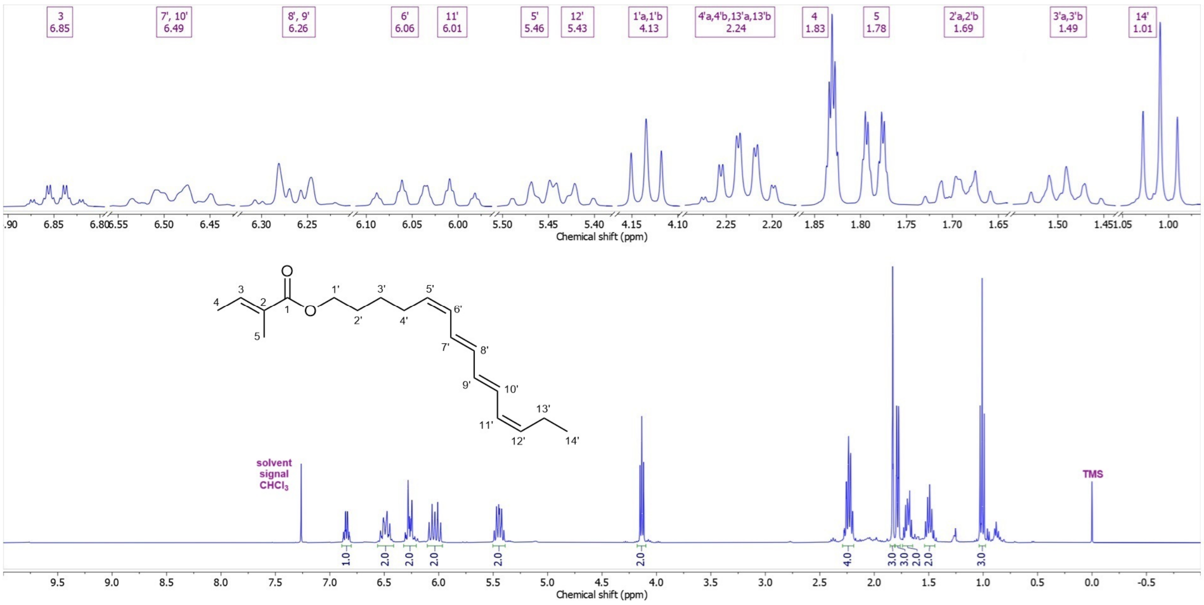

Figure S2. ^{1}H NMR (400 MHz, $CDCl_3$) spectrum of compound **1**

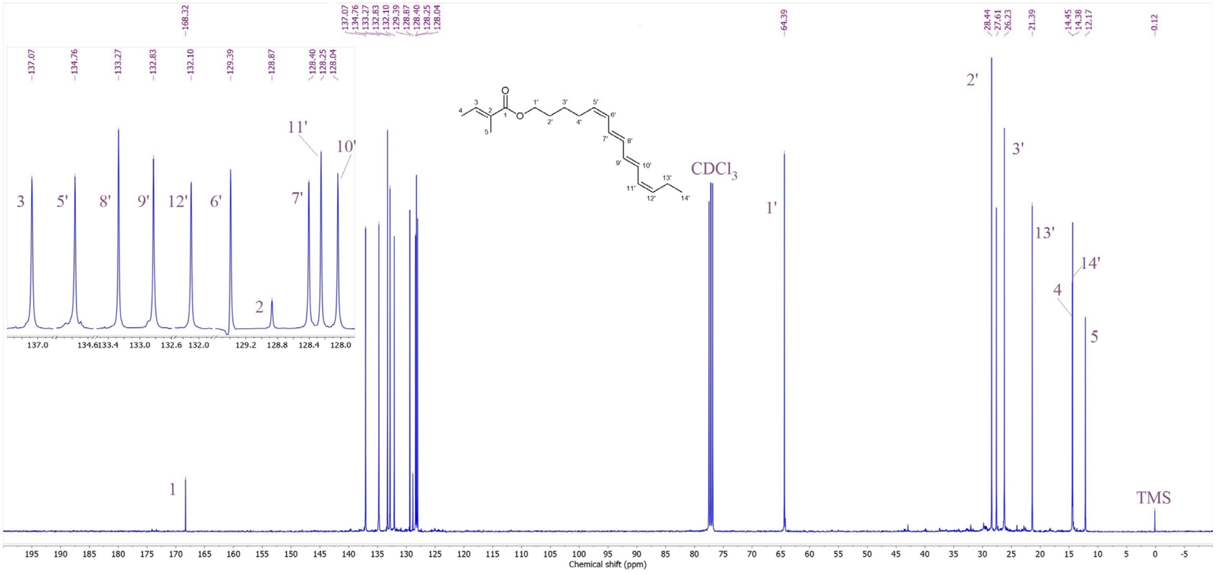

Figure S3. ^{13}C NMR (100.6 MHz, $CDCl_3$) spectrum of compound **1**

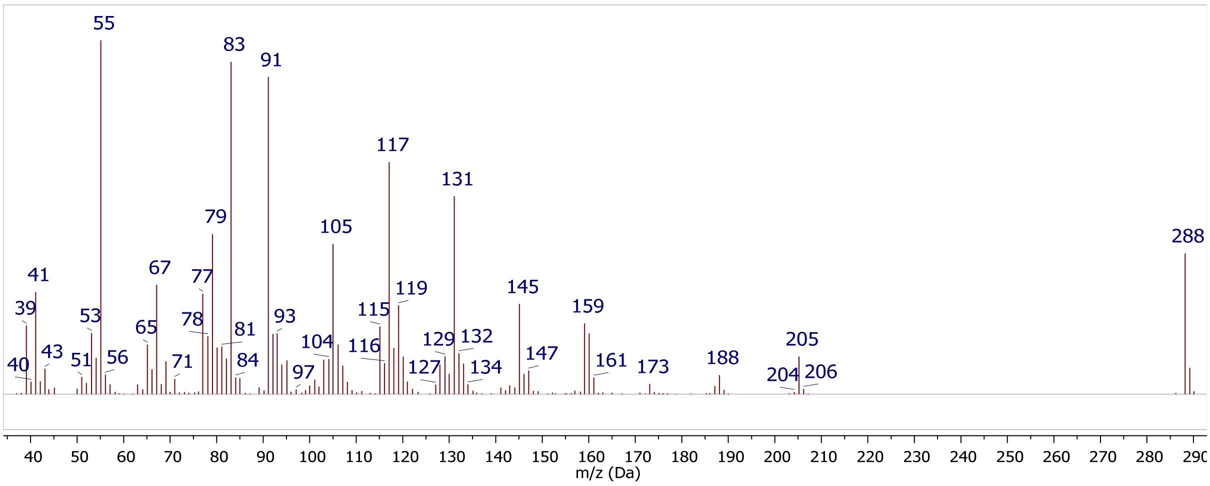

Figure S4. EI (70 eV) mass spectrum of compound **1**

[Mass Spectrum]
Data: MS-BFS-1 Date: 25-Sep-2023 13:18
Instrument : MStation
Sample: -
Note: -
Inlet : Direct Ion Mode : EI+
Spectrum Type : Normal Ion [MF linear]
RT : 6.55 min Scan# : 40 Temp : -
BP: m/z 55 Int. : 85320
Output m/z range : 30 to 295 Cut Level : 0.00%

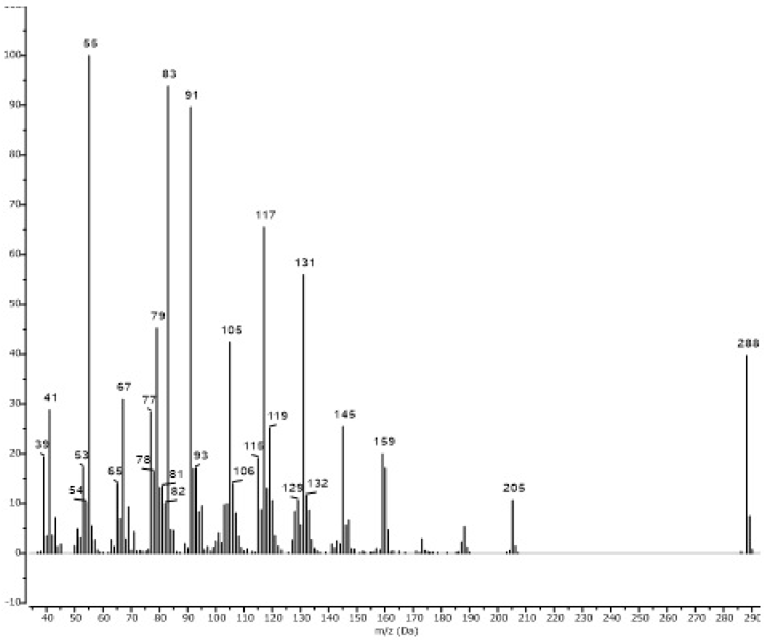

Data: MS-BFS-1 Date: 25-Sep-2023 15:48
Instrument : MStation
Sample: -
Note: -
Inlet : Direct Ion Mode : EI+
RT : 6.55 min Scan# : 40
Elements : C 19/0, H 28/0, O 2/0
Mass Tolerance : 1000ppm, 5mmu if m/z < 5, 50mmu if m/z > 50
Unsaturation (U.S.) : -0.5 -20.0

	Observed m/z	Int%	Err [ppm / mmu]	U.S.	Composition
1	288.2094	39.77	+ 1.7 / + 0.5	6	C19 H28 O2

Figure S5. HRMS spectrum of compound **1**

Table S1. ^{1}H (400 MHz) and ^{13}C (100.6 MHz) NMR spectral data (in $CDCl_3$) of compounds **1** and **2** (NMR parameters are derived from manual iterative full spin analysis)

Compound 1			Compound 2		
**Position**	**δ_H (m, J[1] (Hz), Integral)**	**δ_C (ppm)**	**Position**	**δ_H (m, J (Hz), Integral)**	**δ_C (ppm)**
1	/	168.32	1	/	168.33
2	/	128.87	2	/	128.85
3	6.8468 (qq, $^{3}J_{3,4}$ = 7.1, $^{4}J_{3,5}$ = 1.4, 1 H)	137.07	3	6.8500 (qq, $^{3}J_{3,4}$ = 7.0, $^{4}J_{3,5}$ = 1.5, 1 H)	137.30
4	1.7846 (d, $^{3}J_{3,4}$ = 7.1, 3 H)	14.45	4	1.7860 (dq, $^{3}J_{3,4}$ = 7.0, $^{5}J_{4,5}$ = 1.2, 3 H)	14.39
5	1.8308 (d, $^{4}J_{3,5}$ = 1.4, 3 H)	12.17	5	1.8312 (dq, $^{4}J_{3,5}$ = 1.5, $^{5}J_{4,5}$ = 1.2, 3 H)	11.78
1′a	4.1343 (dd, $^{3}J_{1'a,2'b}$ = 7.7, $^{3}J_{1'a,2'a}$ = 5.5, 1 H)	64.39	1′a	4.1320 (dd, $^{3}J_{1'a,2'b}$ = $^{3}J_{1'a,2'a}$ = 7.0, 1 H)	64.16
1′b	4.1343 (dd, $^{3}J_{1'b,2'a}$ = 7.7, $^{3}J_{1'b,2'b}$ = 5.5, 1 H)		1′b	4.1320 (dd, $^{3}J_{1'b,2'a}$ = $^{3}J_{1'b,2'b}$ = 7.0, 1 H)	
2′a	1.6932 (dddd, $^{3}J_{2'a,3'b}$ = 9.8, $^{3}J_{1'b,2'a}$ = 7.7, $^{3}J_{2'a,3'a}$ = 5.6, $^{3}J_{1'a,2'a}$ = 5.5, 1 H)	28.44	2′a	1.6900 (dddd, $^{3}J_{2'a,3'b}$ = 9.0, $^{3}J_{1'b,2'a}$ = $^{3}J_{1'a,2'a}$ = 7.0, $^{3}J_{2'a,3'a}$ = 5.0, 1 H)	26.15
2′b	1.6932 (dddd, $^{3}J_{2'b,3'a}$ = 9.8, $^{3}J_{1'a,2'b}$ = 7.7, $^{3}J_{2'b,3'b}$ = 5.6, $^{3}J_{1'b,2'b}$ = 5.5, 1 H)		2′b	1.6900 (dddd, $^{3}J_{2'b,3'a}$ = 9.0, $^{3}J_{1'a,2'b}$ = $^{3}J_{1'b,2'b}$ = 7.0, $^{3}J_{2'b,3'b}$ = 5.0, 1 H)	
3′a	1.4922 (dddd, $^{3}J_{2'b,3'a}$ = $^{3}J_{3'a,4'b}$ = 9.8, $^{3}J_{2'a,3'a}$ = $^{3}J_{3'a,4'a}$ = 5.6, 1 H)	26.23	3′a	1.4820 (dddd, $^{3}J_{2'b,3'a}$ = $^{3}J_{3'a,4'b}$ = 9.0, $^{3}J_{2'a,3'a}$ = $^{3}J_{3'a,4'a}$ = 5.0, 1 H)	26.95
3′b	1.4922 (dddd, $^{3}J_{2'a,3'b}$ = $^{3}J_{3'b,4'a}$ = 9.8, $^{3}J_{2'b,3'b}$ = $^{3}J_{3'b,4'b}$ = 5.6, 1 H)		3′b	1.4820 (dddd, $^{3}J_{2'a,3'b}$ = $^{3}J_{3'b,4'a}$ = 9.0, $^{3}J_{2'b,3'b}$ = $^{3}J_{3'b,4'b}$ = 5.0, 1 H)	
4′a	2.2263 (dddd, $^{3}J_{3'b,4'a}$ = 9.8, $^{3}J_{4'a,5'}$ = 7.8, $^{3}J_{3'a,4'a}$ = 5.6, $^{4}J_{4'a,6'}$ = 1.7, 1 H)	27.61	4′a	2.1210 (dddd, $^{3}J_{3'b,4'a}$ = 9.0, $^{3}J_{4'a,5'}$ = 7.3, $^{3}J_{3'a,4'a}$ = 5.0, $^{4}J_{4'a,6'}$ = 1.3, 1 H)	27.58
4′b	2.2263 (dddd, $^{3}J_{3'a,4'b}$ = 9.8, $^{3}J_{4'b,5'}$ = 7.8, $^{3}J_{3'b,4'b}$ = 5.6, $^{4}J_{4'b,6'}$ = 1.7, 1 H)		4′b	2.1210 (dddd, $^{3}J_{3'a,4'b}$ = 9.0, $^{3}J_{4'b,5'}$ = 7.3, $^{3}J_{3'b,4'b}$ = 5.0, $^{4}J_{4'b,6'}$ = 1.3, 1 H)	
5′	5.4562 (dddd, $^{3}J_{5',6'}$ = 11.0, $^{3}J_{4'a,5'}$ = $^{3}J_{4'b,5'}$ = 7.8, $^{4}J_{5',7'}$ = -1.7, 1 H)	134.76	5′	5.3827 (dddd, $^{3}J_{5',6'}$ = 10.8, $^{3}J_{4'a,5'}$ = $^{3}J_{4'b,5'}$ = 7.3, $^{4}J_{5',7'}$ = -0.8, 1 H)	132.03
6′	6.0612 (ddddd, $^{3}J_{5',6'}$ = $^{3}J_{6',7'}$ = 11.0, $^{4}J_{4'a,6'}$ = $^{4}J_{4'b,6'}$ = 1.7, $^{4}J_{6',8'}$ = -0.7, 1 H)	129.39	6′	6.0170 (ddddd, $^{3}J_{6',7'}$ = 11.4, $^{3}J_{5',6'}$ = 10.8, $^{4}J_{4'a,6'}$ = $^{4}J_{4'b,6'}$ = 1.3, $^{4}J_{6',8'}$ = -0.7, 1 H)	129.34
7′	6.5020 (ddddd, $^{3}J_{7',8'}$ = 15.2, $^{3}J_{6',7'}$ = 11.0, $^{4}J_{5',7'}$ = -1.7, $^{4}J_{7',9'}$ = -0.8, $^{5}J_{7',10'}$ = -0.6, 1 H)	128.40	7′	6.3642 (dddd, $^{3}J_{7',8'}$ = 14.8, $^{3}J_{6',7'}$ = 11.4, $^{4}J_{5',7'}$ = -0.8, $^{4}J_{7',9'}$ = -0.7, 1 H)	125.86
8′	6.2700 (dddd, $^{3}J_{7',8'}$ = 15.2, $^{3}J_{8',9'}$ = 12.0, $^{4}J_{8',10'}$ = -0.8, $^{4}J_{6',8'}$ = -0.7, 1 H)	133.27	8′	6.1730 (dddd, $^{3}J_{7',8'}$ = 14.8, $^{3}J_{8',9'}$ = 10.8, $^{4}J_{8',10'}$ = -0.7, $^{4}J_{6',8'}$ = -0.7, 1 H)	132.03
9′	6.2599 (dddd, $^{3}J_{9',10'}$ = 15.2, $^{3}J_{8',9'}$ = 12.0, $^{4}J_{7',9'}$ = -0.8, $^{4}J_{9',11'}$ = -0.7, 1 H)	132.83	9′	6.0980 (ddddd, $^{3}J_{9',10'}$ = 15.0, $^{3}J_{8',9'}$ = 10.8, $^{4}J_{7',9'}$ = -0.7, $^{4}J_{9',11'a}$ = $^{4}J_{9',11'b}$ = 1.3, 1 H)	130.62
10′	6.4780 (ddddd, $^{3}J_{9',10'}$ = 15.2, $^{3}J_{10',11'}$ = 11.0, $^{4}J_{10',12'}$ = 1.0, $^{4}J_{8',10'}$ = -0.8, $^{5}J_{7',10'}$ = -0.6, 1 H)	128.04	10′	5.7092 (dddd, $^{3}J_{9',10'}$ = 15.0, $^{3}J_{10',11'a}$ = $^{3}J_{10',11'b}$ = 6.9, $^{4}J_{8',10'}$ = -0.7, 1 H)	135.60
11′	6.0093 (ddddd, $^{3}J_{10',11'}$ = $^{3}J_{11',12'}$ = 11.0, $^{4}J_{11',13'a}$ = $^{4}J_{11',13'b}$ = 1.6, $^{4}J_{9',11'}$ = -0.7, 1 H)	128.25	11′a	2.1050 (dddd, $^{3}J_{10',11'a}$ = 6.9, $^{3}J_{11'a,12'a}$ = $^{3}J_{11'a,12'b}$ = 7.2, $^{4}J_{9',11'a}$ = 1.3, 1 H)	32.64
			11′b	2.1050 (dddd, $^{3}J_{10',11'b}$ = 6.9, ^{3} $^{3}J_{11'b,12'a}$ = $^{3}J_{11'b,12'b}$ = 7.2, $^{4}J_{9',11'b}$ = 1.3, 1 H)	
12′	5.4346 (dddd, $^{3}J_{11',12'}$ = 11.0, $^{3}J_{12',13'a}$ = $^{3}J_{12',13'b}$ = 7.8, $^{4}J_{10',12'}$ = 1.0, 1 H)	132.10	12′a	1.2930 (dddd, $^{3}J_{12a',13'b}$ = 9.0, $^{3}J_{11'a,12'a}$ = $^{3}J_{11'b,12'a}$ = 7.2, $^{3}J_{12a',13'a}$ = 5.0, 1 H)	31.58
			12′b	1.2930 (dddd, $^{3}J_{12b',13'a}$ = 9.0, $^{3}J_{11'a,12'b}$ = $^{3}J_{11'b,12'b}$ = 7.2, $^{3}J_{12b',13'b}$ = 5.0, 1 H)	
13′a	2.2367 (ddd, $^{3}J_{12',13'a}$ = 7.8, $^{3}J_{13'a,14'}$ = 7.5, $^{4}J_{11',13'a}$ = 1.6, 1 H)	21.39	13′a	1.3670 (ddd, $^{3}J_{12b',13'a}$ = 9.0, $^{3}J_{13'a, 14'}$ = 7.0, $^{3}J_{12a',13'a}$ = 5.0, 1 H)	22.55
13′b	2.2367 (ddd, $^{3}J_{12',13'b}$ = 7.8, $^{3}J_{13'b,14'}$ = 7.5, $^{4}J_{11',13'b}$ = 1.6, 1 H)		13′b	1.3670 (ddd, $^{3}J_{12a',13'b}$ = 9.0, $^{3}J_{13'b,14'}$ = 7.0, $^{3}J_{12b',13'b}$ = 5.0, 1 H)	
14′	1.0092 (t, $^{3}J_{13'a, 14'}$ = $^{3}J_{13'b,14'}$ = 7.5, 3 H)	14.38	14′	0.8964 (t, $^{3}J_{13'a, 14'}$ = $^{3}J_{13'b,14'}$ = 7.0, 3 H)	14.07

[1] Coupling constant values were initially inferred from ^{1}H homoselective decoupling NMR experiments and afterward refined through a manual iterative full spin analysis. For details, cf. Experimental part.

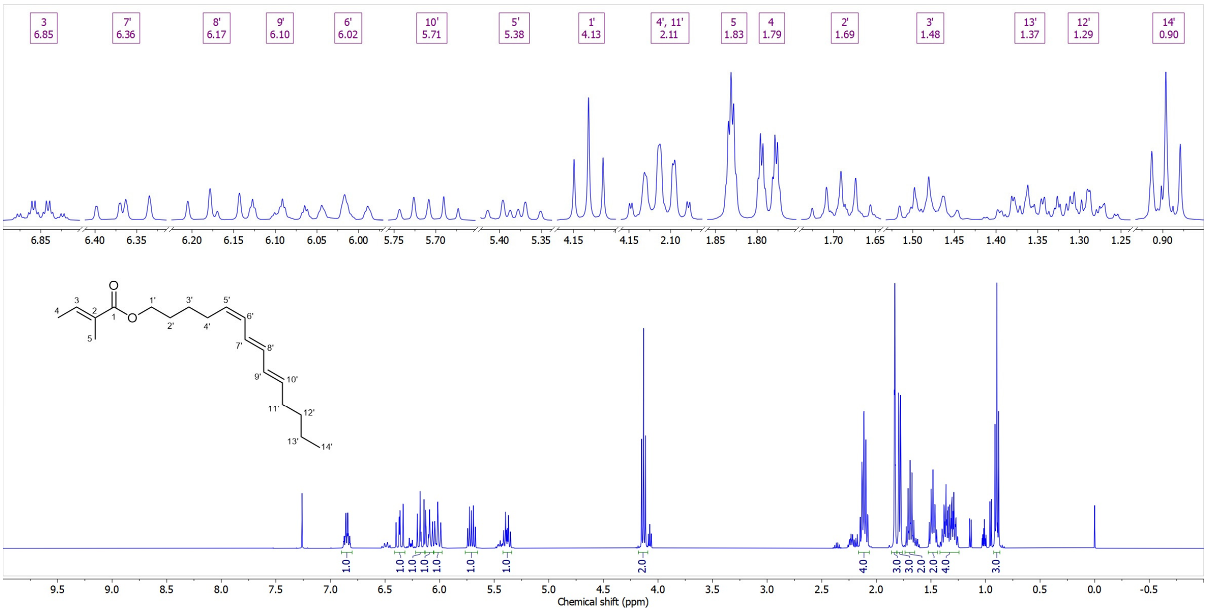

Figure S6. ^{1}H NMR (400 MHz, $CDCl_3$) spectrum of compound **2**

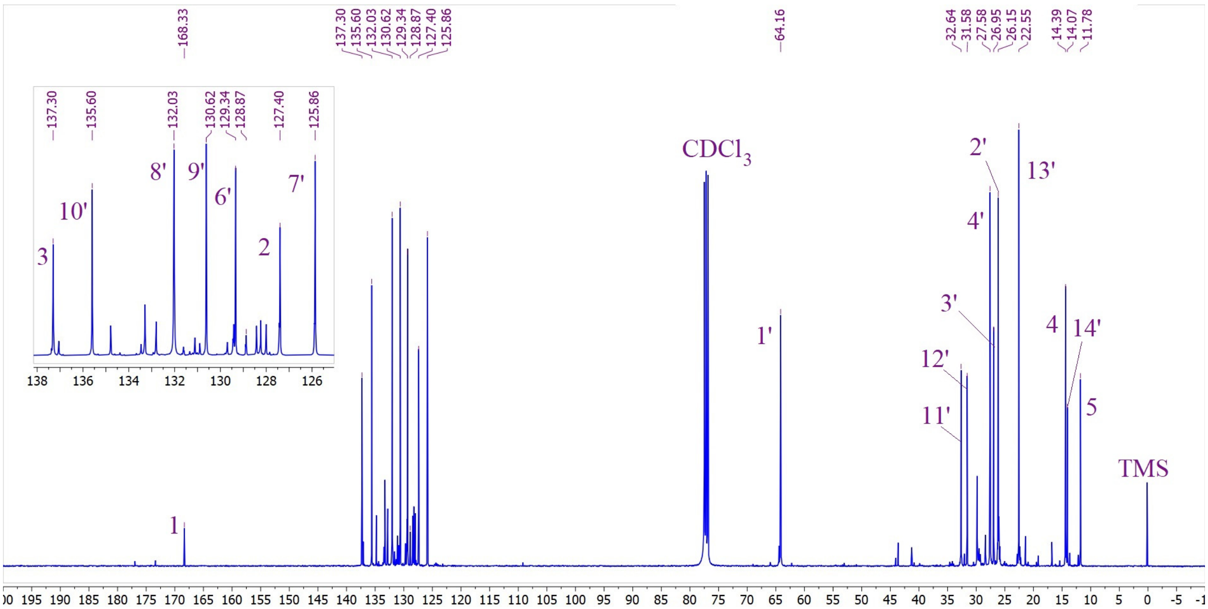

Figure S7. ^{13}C NMR (100.6 MHz, $CDCl_3$) spectrum of compound **2**

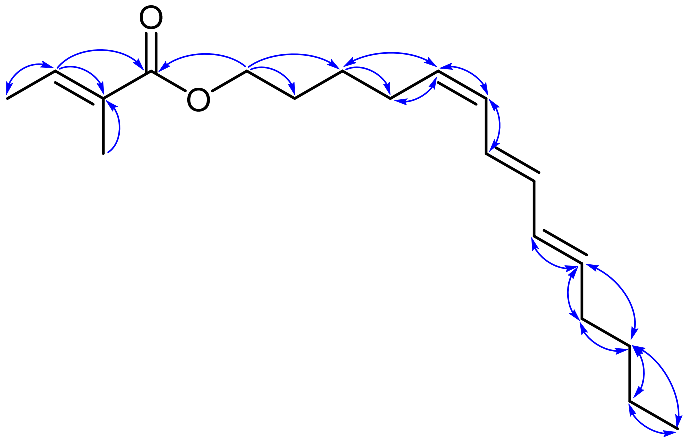

Figure S8. Important HMBC interactions of compound **2**

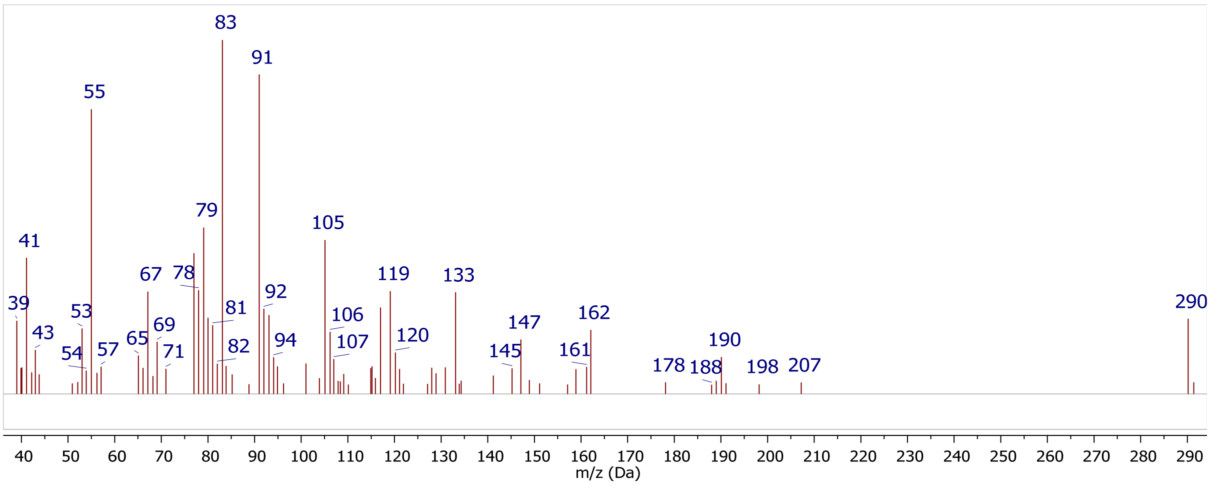

Figure S9. EI (70 eV) mass spectrum of compound **2**

[Mass Spectrum]
Data: MS-BFS-2 Date: 1-Oct-2023 10:39
Instrument : MStation
Sample: -
Note: -
Inlet : Direct Ion Mode : EI+
Spectrum Type : Normal Ion [MF linear]
RT : 6.28 min Scan# : 38 Temp : -
BP: m/z 83 Int. : 5732
Output m/z range : 35 to 295 Cut Level : 0.00%

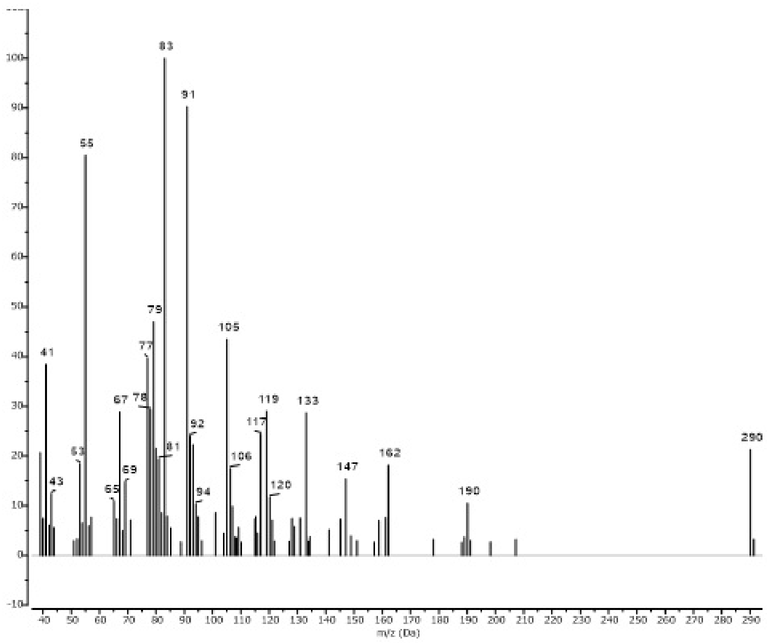

Data: MS-BFS-2 Date: 1-Oct-2023 11:58
Instrument : MStation
Sample: -
Note: -
Inlet : Direct Ion Mode : EI+
RT : 6.28 min Scan# : 38
Elements : C 19/0, H 30/0, O 2/0
Mass Tolerance : 1000ppm, 5mmu if m/z < 5, 50mmu if m/z > 50
Unsaturation (U.S.) : -0.5 -20.0

	Observed m/z	Int%	Err [ppm / mmu]	U.S.	Composition
1	290.2251	21.28	+ 1.7 / + 0.5	5	C19 H30 O2

Figure S10. HRMS spectrum of compound **2**

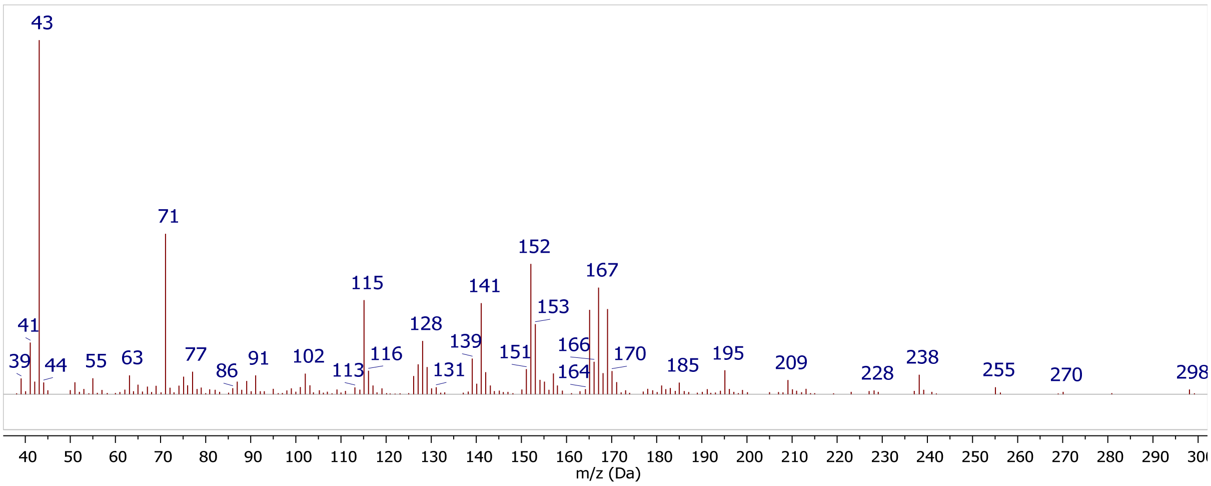

Figure S11. EI (70 eV) mass spectrum of compound **6**

[Mass Spectrum]
Data: MS-BFS-6 Date: 28-Oct-2023 15:28
Instrument : MStation
Sample: -
Note: -
Inlet : Direct Ion Mode : EI+
Spectrum Type : Normal Ion [MF linear]
RT : 7.76 min Scan# : 49 Temp : -
BP: m/z 43 Int. : 75544
Output m/z range : 35 to 305 Cut Level : 0.00%

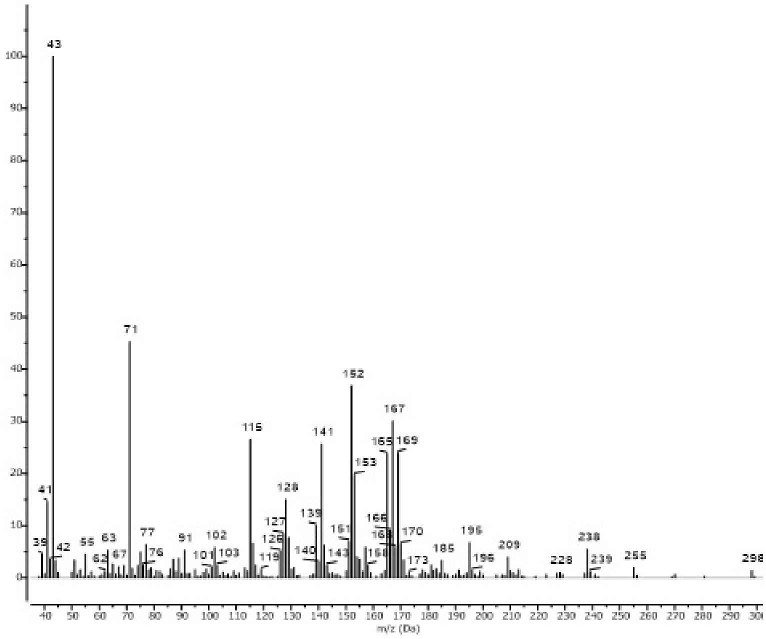

Data: MS-BFS-6 Date: 28-Oct-2023 17:53
Instrument : MStation
Sample: -
Note: -
Inlet : Direct Ion Mode : EI+
RT : 7.76 min Scan# : 49
Elements : C 19/0, H 22/0, O 3/0
Mass Tolerance : 1000ppm, 5mmu if m/z < 5, 50mmu if m/z > 50
Unsaturation (U.S.) : -0.5 -20.0

	Observed m/z	Int%	Err [ppm / mmu]	U.S.	Composition
1	298.1574	2.1	+ 1.7 / + 0.5	9	C19 H22 O3

Figure S12. HRMS spectrum of compound **6**

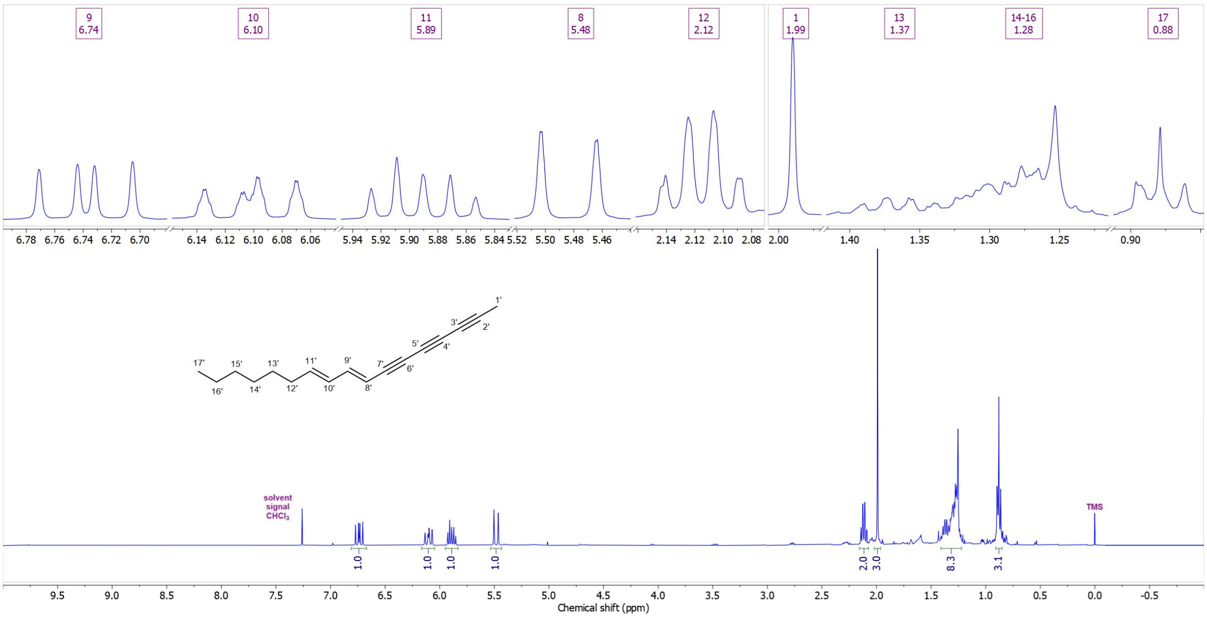

Figure S13. ^{1}H NMR (400 MHz, $CDCl_3$) spectrum of compound **8**

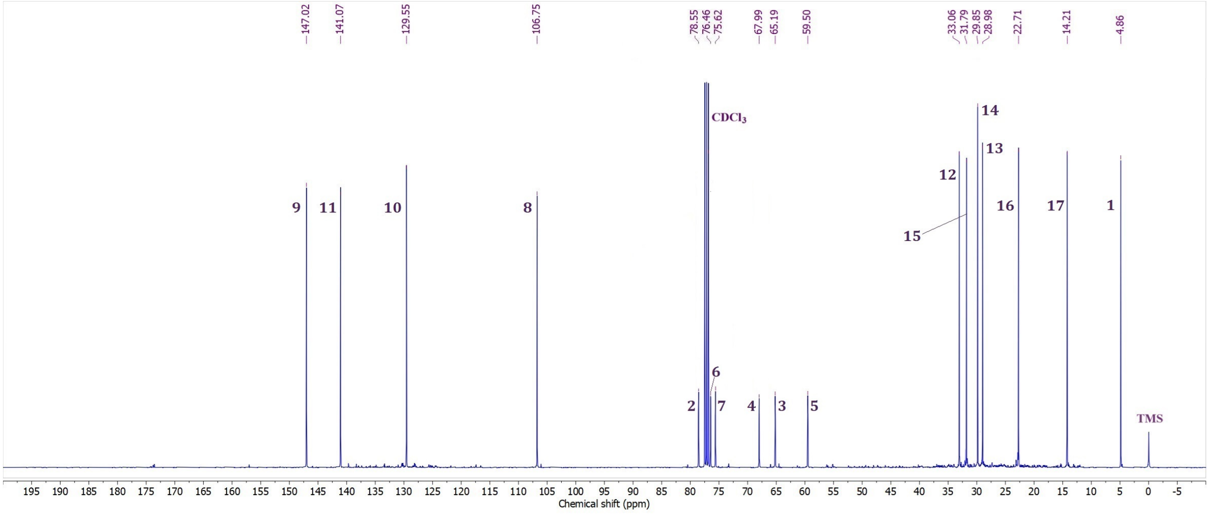

Figure S14. ^{13}C NMR (100.6 MHz, $CDCl_3$) spectrum of compound **8**

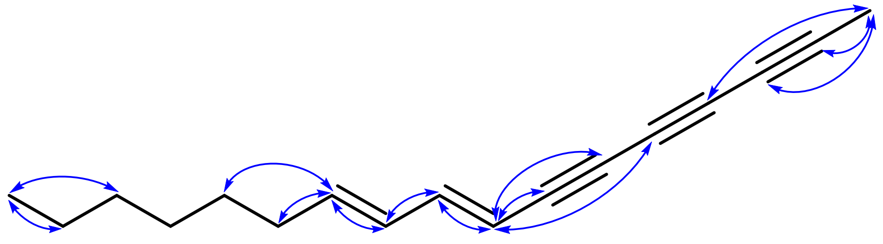

Figure S15. Important HMBC interactions of compound **8**

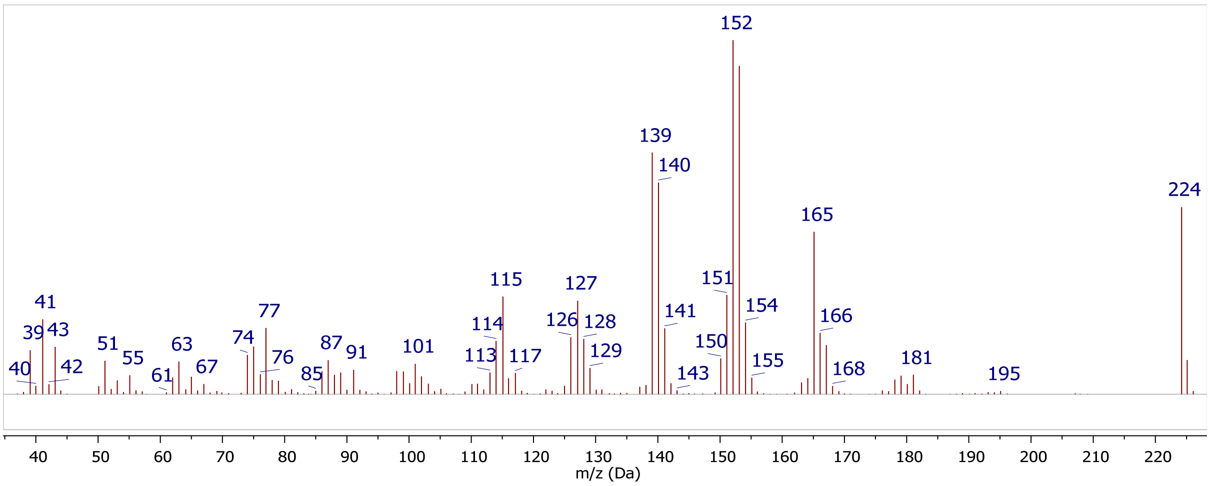

Figure S16. EI (70 eV) mass spectrum of compound **8**

[Mass Spectrum]
Data: MS-BFG-8 Date: 15-Aug-2023 18:37
Instrument : MStation
Sample: -
Note: -
Inlet : Direct Ion Mode : EI+
Spectrum Type : Normal Ion [MF linear]
RT : 6.01 min Scan# : 36 Temp : -
BP: m/z 152 Int. : 162752
Output m/z range : 35 to 230 Cut Level : 0.00%

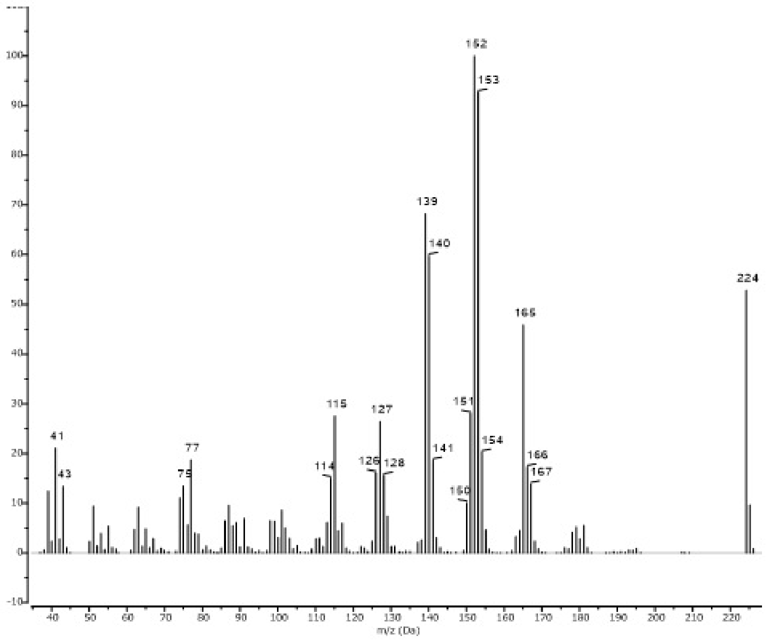

Data: MS-BFG-8 Date: 15-Aug-2023 21:09
Instrument : MStation
Sample: -
Note: -
Inlet : Direct Ion Mode : EI+
RT : 6.01 min Scan# : 36
Elements : C 17/0, H 20/0
Mass Tolerance : 1000ppm, 5mmu if m/z < 5, 50mmu if m/z > 50
Unsaturation (U.S.) : -0.5 -20.0

	Observed m/z	Int%	Err [ppm / mmu]	U.S.	Composition
1	224.1570	52.90	+ 2.2 / + 0.5	8	C17 H20

Figure S17. HRMS spectrum of compound **8**

Table S2. ^{1}H (400 MHz) and ^{13}C (100.6 MHz) NMR spectral data (in $CDCl_3$) of compound **8** (NMR parameters are derived from manual iterative full spin analysis)

Position	δ_H (m, J[1] (Hz), Integral)	δ_C (ppm)
1	1.9898 (s, 3 H)	4.86
2	/	78.55
3	/	65.19
4	/	67.99
5	/	59.50
6	/	76.46
7	/	75.62
8	5.4839 (ddd, $^{3}J_{8,9}$ = 15.6, $^{4}J_{8,10}$ = 0.7, $^{5}J_{8,11}$ = 0.6, 1 H)	106.75
9	6.7374 (ddd, $^{3}J_{8,9}$ = 15.6, $^{3}J_{9,10}$ = 10.9, $^{4}J_{9,11}$ = 0.6, 1 H)	147.02
10	6.1009 (ddtd, $^{3}J_{10,11}$ = 15.1, $^{3}J_{9,10}$ = 10.9, $^{4}J_{10,12}$ = 1.3, $^{4}J_{8',10'}$ = 0.7, 1 H)	129.55
11	5.8917 (dtdd, $^{3}J_{10,11}$ = 15.1, $^{3}J_{11,12}$ = 7.2, $^{4}J_{9,11}$ = $^{5}J_{8',11'}$ = 0.6, 1 H)	141.07
12	2.1153 (m, $^{3}J_{11,12}$ = 7.2, $^{4}J_{10,12}$ = 1.3, 2 H)	33.06
13[2]	1.3723 (overlapped multiplets, 2 H)	28.98
14[2]	1.3070 (overlapped multiplets, 2 H)	29.85
15[2]	1.2550 (overlapped multiplets, 2 H)	31.79
16[2]	1.2880 (m, $^{3}J_{16,17}$ = 7.2, 2 H)	22.71
17	0.8790 (t, $^{3}J_{16,17}$ = 7.2, 3 H)	14.21

[1] Coupling constant values were initially inferred from ^{1}H homoselective decoupling NMR experiments and afterward refined through a manual iterative full spin analysis. For details, cf. Experimental part. [2] Severely overlapping multiplets; the signals were not simulated.

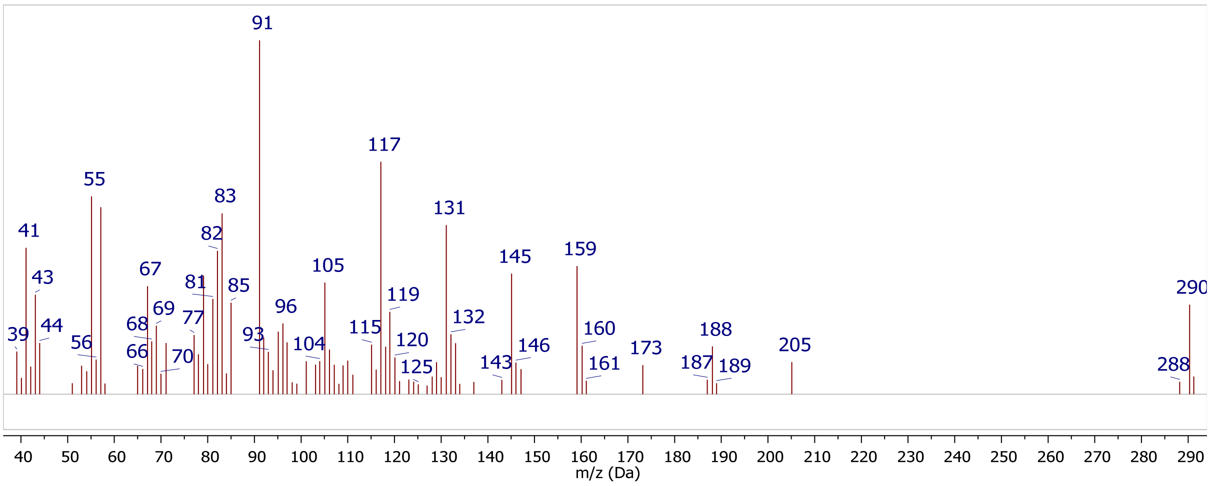

Figure S18. EI (70 eV) mass spectrum of praealtaester B

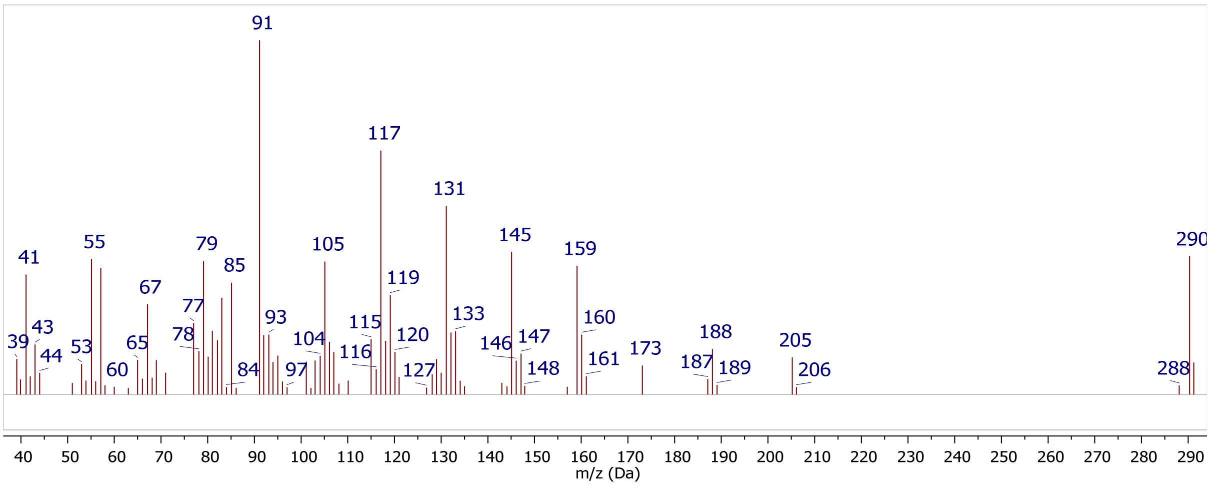

Figure S19. EI (70 eV) mass spectrum of praealtaester B stereoisomer 1

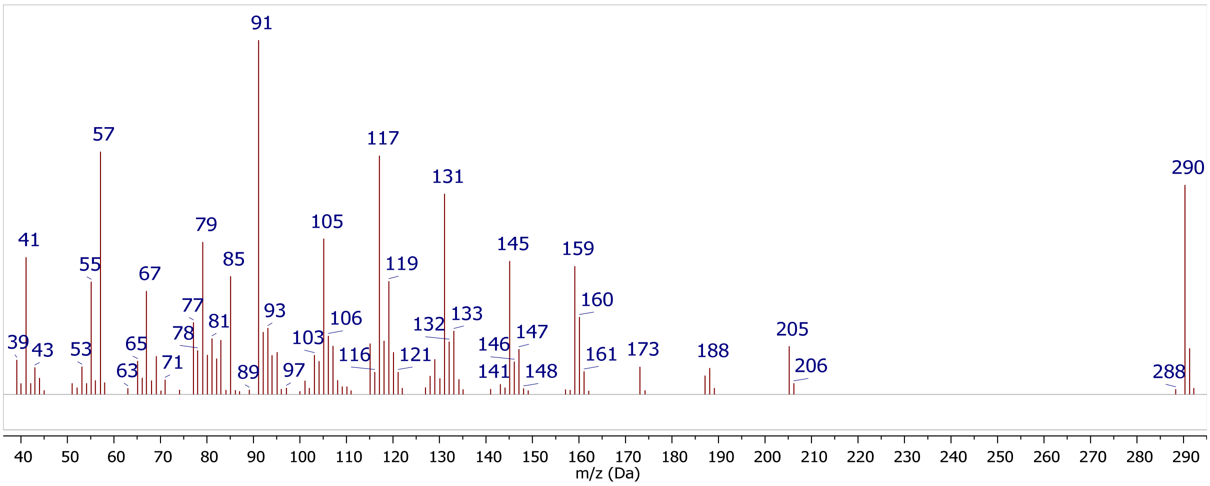

Figure S20. EI (70 eV) mass spectrum of praealtaester B stereoisomer 2

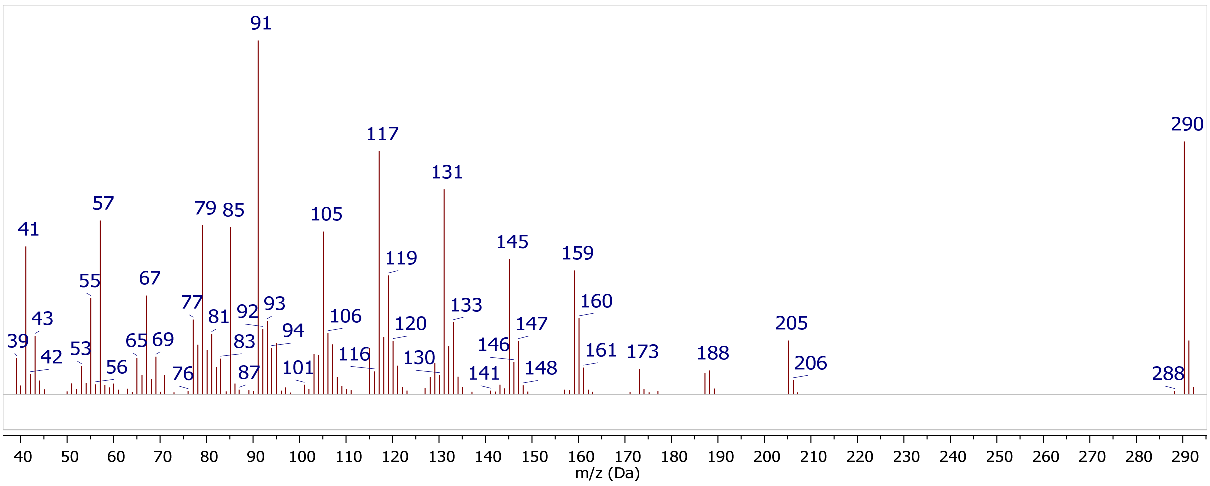

Figure S21. EI (70 eV) mass spectrum of praealtaester B stereoisomer 3

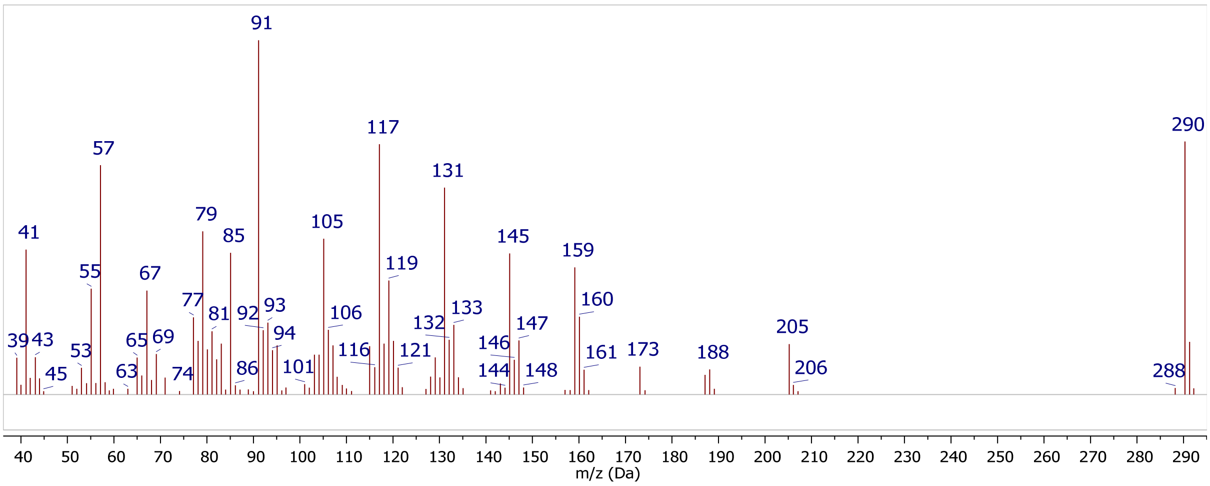

Figure S22. EI (70 eV) mass spectrum of praealtaester B stereoisomer 4

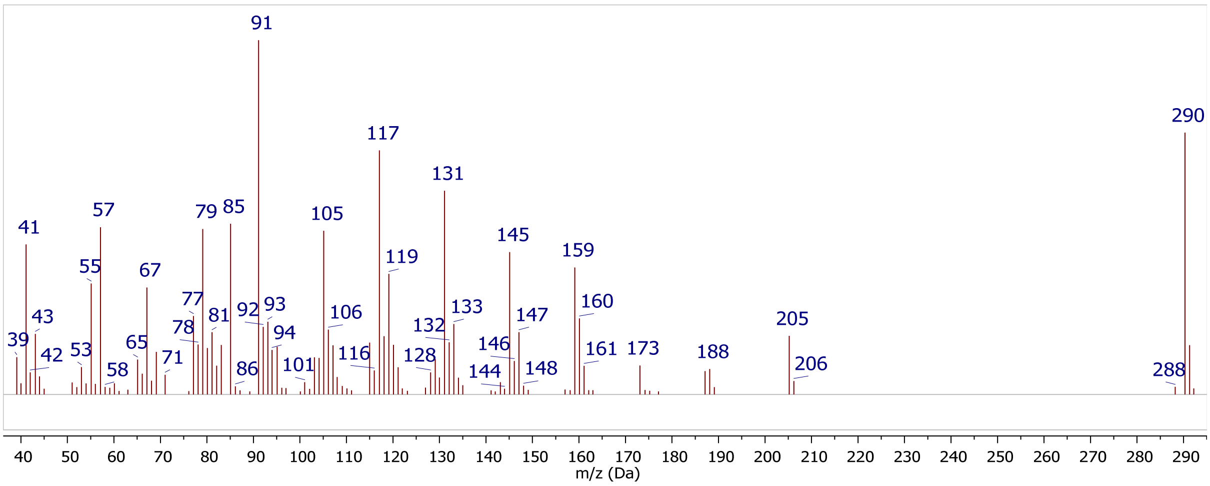

Figure S23. EI (70 eV) mass spectrum of praealtaester B stereoisomer 5

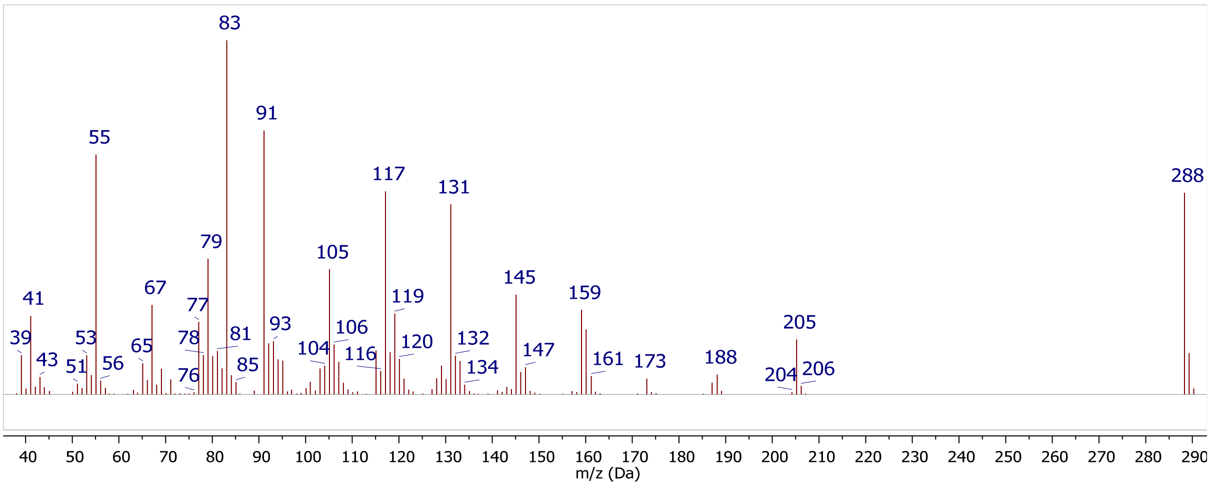

Figure S24. EI (70 eV) mass spectrum of compound **1** stereoisomer 1

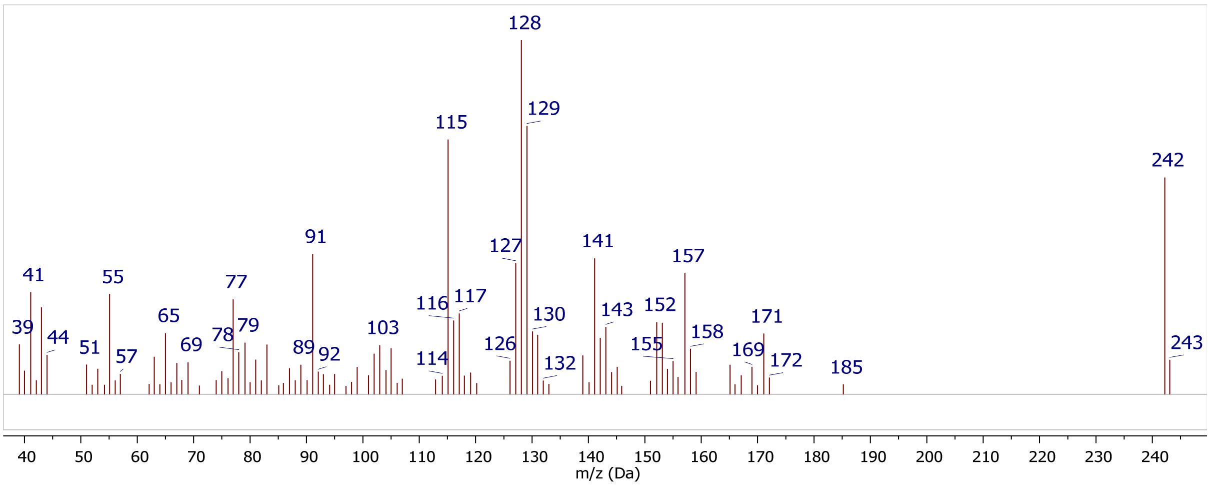

Figure S25. EI (70 eV) mass spectrum of compound **15** stereoisomer 1

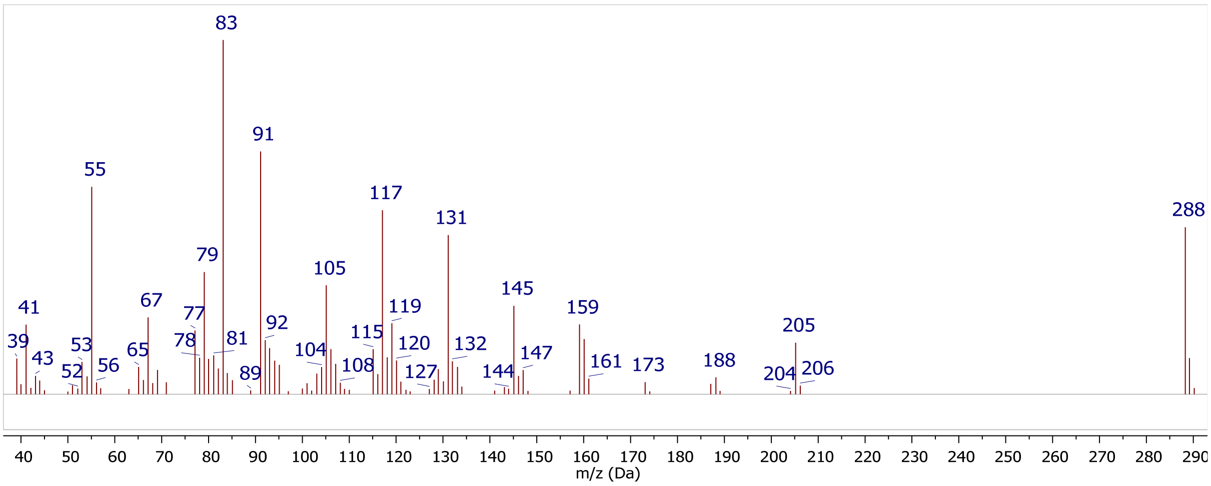

Figure S26. EI (70 eV) mass spectrum of compound **1** stereoisomer 2

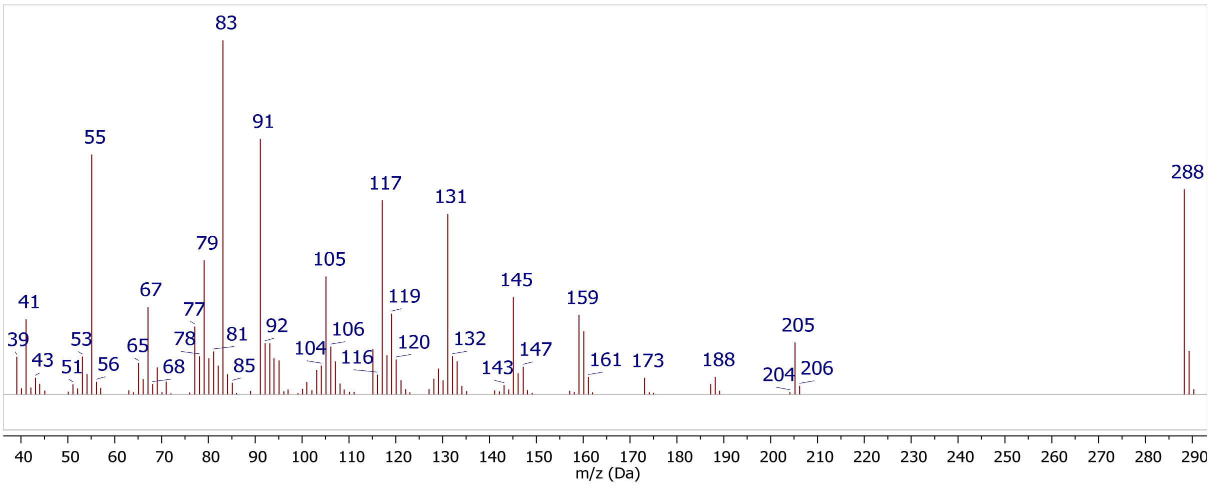

Figure S27. EI (70 eV) mass spectrum of compound **1** stereoisomer 3

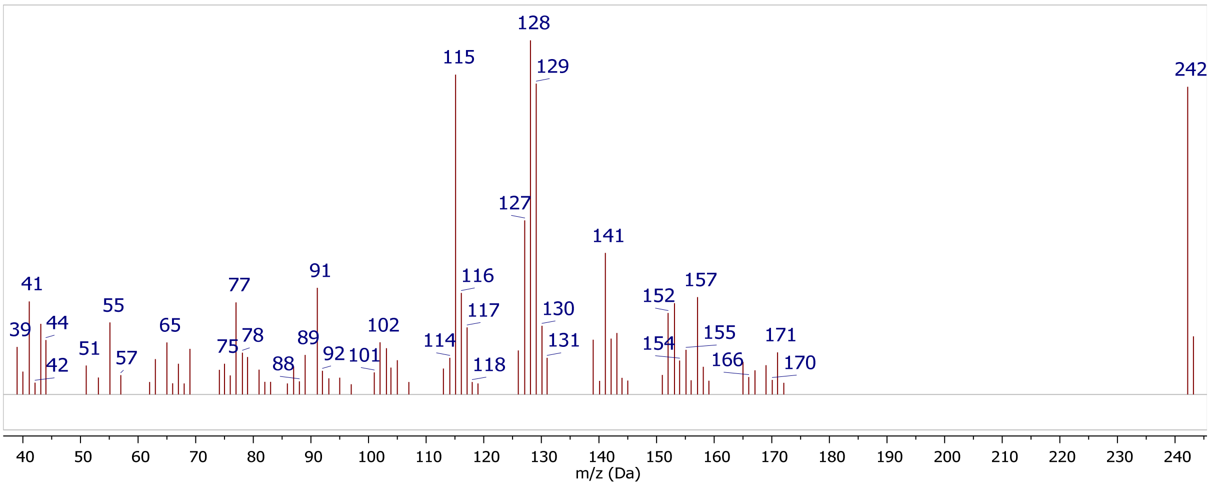

Figure S28. EI (70 eV) mass spectrum of compound **15** stereoisomer 2

Table S3. ^{1}H (400 MHz) and ^{13}C (100.6 MHz) NMR spectral data (in $CDCl_3$) of compound **5** (NMR parameters are derived from manual iterative full spin analysis)

**Position**	**δ_H (m, J[1] (Hz), Integral)**	**δ_C (ppm)**
1	/	168.38
2	/	128.90
3	6.8454 (qq, $^{3}J_{3,4}$ = 7.1, $^{4}J_{3,5}$ = 1.5, 1 H)	136.91
4	1.7862 (dq, $^{3}J_{3,4}$ = 7.1, $^{5}J_{4,5}$ = 1.2, 3 H)	14.43
5	1.8314 (dq, $^{4}J_{3,5}$ = 1.5, $^{5}J_{4,5}$ = 1.2, 3 H)	12.15
1′a	4.1138 (dd, $^{3}J_{1'a,2'b}$ = $^{3}J_{1'a,2'a}$ = 7.0, 1 H)	64.67
1′b	4.1138 (dd, $^{3}J_{1'b,2'a}$ = $^{3}J_{1'b,2'b}$ = 7.0, 1 H)	
2′a	1.6729 (dddd, $^{3}J_{2'a,3'b}$ = 9.0, $^{3}J_{1'b,2'a}$ = $^{3}J_{1'a,2'a}$ = 7.0, $^{3}J_{2'a,3'a}$ = 5.0, 1 H)	28.81
2′b	1.6729 (dddd, $^{3}J_{2'b,3'a}$ = 9.0, $^{3}J_{1'a,2'b}$ = $^{3}J_{1'b,2'b}$ = 7.0, $^{3}J_{2'b,3'b}$ = 5.0, 1 H)	
3′	1.36 (overlapped multiplets, 1 H)	26.13
4′-7′	1.25-1.40 (overlapped multiplets, 8 H)	28.32-29.50
8′	2.17 (overlapped multiplets, 2 H)	27.94
9′	5.3927 (dtd, $^{3}J_{9',10'}$ = 10.6, $^{3}J_{8',9'}$ = 7.7, $^{4}J_{9',11'}$ = -0.8, 1 H)	131.99
10′	5.9904 (ddtd, $^{3}J_{10',11'}$ = 11.4, $^{3}J_{9',10'}$ = 10.6, $^{4}J_{8',10'}$ = -1.4, $^{4}J_{10',12'}$ = -0.7, 1 H)	128.80
11′	6.3785 (dddd, $^{3}J_{11',12'}$ = 14.8, $^{3}J_{10',11'}$ = 11.4, $^{4}J_{9',11'}$ = -0.8, $^{4}J_{11',13'}$ = -0.7, 1 H)	126.07
12′	6.1630 (dddd, $^{3}J_{11',12'}$ = 14.8, $^{3}J_{12',13'}$ = 10.8, $^{4}J_{12',14'}$ = $^{4}J_{10',12'}$ = -0.7, 1 H)	132.91
13′	6.1050 (ddtd, $^{3}J_{13',14'}$ = 15.0, $^{3}J_{12',13'}$ = 10.8, $^{4}J_{13',15'}$ = 1.3, $^{4}J_{11',13'}$ = -0.7, 1 H)	130.66
14′	5.6992 (dtd, $^{3}J_{13',14'}$ = 15.0, $^{3}J_{14',15'}$ = 6.9, $^{4}J_{12',14'}$ = -0.7, 1 H)	135.29
15′	2.1050 (overlapped multiplets, 2 H)	32.62
16′	1.36 (overlapped multiplets, 2H)	31.58
17′	1.33 (overlapped multiplets, 2H)	22.35
18′	0.8942 (t, $^{3}J_{17', 18'}$ = 3 H)	14.06

[1] Coupling constant values were initially inferred from ^{1}H homoselective decoupling NMR experiments and afterward refined through a manual iterative full spin analysis. For details, cf. Experimental part. [2] Severely overlapping multiplets; the signals were not simulated.
